# An improved Red-billed blue magpie feature selection algorithm for medical data processing

**DOI:** 10.1371/journal.pone.0324866

**Published:** 2025-05-22

**Authors:** Chenyi Zhu, Zhiyi Wang, Yinan Peng, Wenjun Xiao

**Affiliations:** 1 School of Mechanical and Automotive Engineering, Jinken College of Technology, China; 2 College of Civil Aviation, Nanjing University of Aeronautics and Astronautics, China; 3 Shanghai Palmim Information Technology Ltd, China; 4 Hunan Labor And Human Resources Vocational College, School of Intelligent Equipment Manufacturing, China; SR University, INDIA

## Abstract

Feature selection is a crucial preprocessing step in the fields of machine learning, data mining and pattern recognition. In medical data analysis, the large number and complexity of features are often accompanied by redundant or irrelevant features, which not only increase the computational burden, but also may lead to model overfitting, which in turn affects its generalization ability. To address this problem, this paper proposes an improved red-billed blue magpie algorithm (IRBMO), which is specifically optimized for the feature selection task, and significantly improves the performance and efficiency of the algorithm on medical data by introducing multiple innovative behavioral strategies. The core mechanisms of IRBMO include: elite search behavior, which improves global optimization by guiding the search to expand in more promising directions; collaborative hunting behavior, which quickly identifies key features and promotes collaborative optimization among feature subsets; and memory storage behavior, which leverages historically valid information to improve search efficiency and accuracy. To adapt to the feature selection problem, we convert the continuous optimization algorithm to binary form via transfer function, which further enhances the applicability of the algorithm. In order to comprehensively verify the performance of IRBMO, this paper designs a series of experiments to compare it with nine mainstream binary optimization algorithms. The experiments are based on 12 medical datasets, and the results show that IRBMO achieves optimal overall performance in key metrics such as fitness value, classification accuracy and specificity. In addition, compared with nine existing feature selection methods, IRBMO demonstrates significant advantages in terms of fitness value. To further enhance the performance, this paper also constructs the V2IRBMO variant by combining the S-shaped and V-shaped transfer functions, which further enhances the robustness and generalization ability of the algorithm. Experiments demonstrate that IRBMO exhibits high efficiency, generality and excellent generalization ability in feature selection tasks. In addition, used in conjunction with the KNN classifier, IRBMO significantly improves the classification accuracy, with an average accuracy improvement of 43.89% on 12 medical datasets compared to the original Red-billed Blue Magpie algorithm. These results demonstrate the potential and wide applicability of IRBMO in feature selection for medical data.

## 1. Introduction

With the full arrival of the data era, data has been deeply integrated into people’s lives and become an indispensable key element. Among them, medical data [1] has attracted much attention due to its rich life and health information and its important role in disease exploration, diagnosis and treatment. However, the high-dimensional complexity and diversity of healthcare data pose a serious challenge of “dimensionality disaster” [2]. The presence of high-dimensional features not only complicates data processing but can also diminish the accuracy and reliability of the analysis outcomes. Conventional approaches often fall short when handling high-dimensional medical data, making it challenging to fully uncover its underlying potential. Therefore, optimizing the processing and analysis of high-dimensional medical data has become a key issue in the field of data science [3]. Data mining [4] techniques extract useful information and knowledge from massive data through statistics, machine learning and artificial intelligence to support decision making, predict trends and discover potential value. However, massive subsets of features may exist in high-dimensional data, making it impractical to find the optimal subset directly. In this context, data dimensionality reduction techniques have emerged, which are mainly categorized into two methods: feature extraction [5] and feature selection [6].

Feature selection [6] significantly improves the performance, interpretability, and computational efficiency of machine learning models by identifying and retaining the most valuable features in the data, which is particularly important in areas such as medical diagnosis and fault prediction. Different application scenarios have different requirements for feature selection, which requires the development of customized methods to ensure high accuracy and minimize information loss based on the specific problem and data characteristics. Feature selection [7] eliminates redundant and irrelevant features through statistical analysis, model evaluation, or algorithmic mechanisms to reduce noise and improve the generalization ability of the model. Its methods are mainly categorized into three types: filtered [8], embedded [9] and wrapped [10], each with its own advantages and applicable scenarios, providing diversified solutions for high-dimensional data processing. Filtered feature selection [8] screens key features by measuring the statistical correlation or dependence between features and target variables, e.g., by utilizing statistical metrics such as correlation coefficient, variance and mutual information. The method is computationally simple, efficient in execution, and particularly good at handling large-scale datasets. However, its limitation is that evaluating each feature in isolation ignores the potential interaction effects among features, which may lead to the erroneous deletion of some features that perform well in a particular combination. Embedded feature selection [9], on the other hand, integrates the feature selection mechanism into the training process of the learner, and leverages the machine learning algorithm’s own properties (e.g., regularization techniques) to automatically filter the optimal subset of features. This approach performs well when dealing with linear relationship features, but it is weak when facing nonlinear relationships, and its interpretability is relatively weak.

In contrast, packaged feature selection [11] stands out with its unique optimization perspective. It regards feature selection as a complex optimization problem, generates diverse feature subsets through systematic search, and utilizes performance metrics of machine learning models to accurately evaluate the effectiveness of these subsets. This process begins with the selection of an appropriate evaluation model, followed by the generation of a series of feature subsets using efficient strategies such as recursive search, and the rigorous validation of their performance on an independent validation or test set. Compared with the previous two approaches, wrapper feature selection [12] shows significant advantages: it directly evaluates the model performance as a benchmark, and the selected subset of features can be more relevant to the actual application requirements; at the same time, by comprehensively exploring the feature space, the wrapper approach is able to discover the feature combinations that are optimal for the performance of a specific model, which is undoubtedly a great advantage for dealing with datasets that have complex interactions between the features and have very high requirements on the performance of the model [13].

Packed feature selection, although effective in the field of feature optimization, still faces the challenges of high computational cost and the possibility of not always finding the globally optimal feature combination [13]. To cope with these challenges, metaheuristic algorithms [14] have emerged as an important adjunct to wrapper feature selection with their excellent performance in complex optimization tasks. With powerful search capabilities and flexible optimization frameworks, meta-heuristic algorithms are able to efficiently explore large feature spaces under complex constraints, and their integration into the packaged feature selection framework not only significantly reduces the burden of computational resources, but also significantly improves the efficiency and accuracy of feature selection [15]. These algorithms can efficiently identify an optimal or near-optimal feature subset through a smart search strategy, thereby minimizing computational resource waste. Simultaneously, these algorithms exhibit strong compatibility with the feature selection evaluation framework, ensuring that the chosen features significantly decrease computational overhead while preserving the model’s efficacy [16]. This combination strategy provides an innovative idea for solving the feature selection problem for large-scale datasets, and shows a broad application prospect especially in high-dimensional complex fields such as medical engineering [17]. This method enhances the overall efficiency of feature selection while ensuring the model’s reliability and stability in real-world applications. The synergy between meta-heuristic algorithms and packed feature selection opens up a new way for feature selection in large-scale data processing with high efficiency and accuracy, fully demonstrating its great potential and value in the field of data science [16]. Specifically, Ghaemi et al. [18] introduced a forest optimization algorithm for feature selection, aiming to identify the most relevant features from a dataset. Samieiyan et al. [19] developed a feature selection method based on the crow search algorithm, which enhances the exploration phase and effectively reduces dataset dimensionality. Jia et al. [20] proposed three hybrid algorithms that combine the Seagull Optimization Algorithm (SOA) with Thermal Exchange Optimization (TEO) to address the feature selection challenge. Xu et al. [21] presented various Binary Arithmetic Optimization Algorithms (BAOA), each using different strategies to optimize feature selection. Too et al. [22] put forward an improved Competitive Genetic Algorithm, along with a faster version, to enhance the efficiency of genetic algorithms in feature selection.

Metaheuristic algorithms, while showing great potential in the field of feature selection, still face significant challenges [23]. The main challenge lies in striking a balance between exploration and exploitation, with the issue of parameter tuning further limiting performance. In addition, some algorithms neglect computational efficiency in the pursuit of innovation, making it difficult to cope with complex real-world problems. Therefore, when applying them to feature selection, it is necessary to optimize them for specific problems in order to improve the practical performance. Based on the “no free lunch theorem” [24], i.e., there is no universal algorithm that can be applied to all optimization problems, researchers have proposed diversified improvement strategies for different feature selection requirements. These strategies optimize the search mechanism and strike a balance between efficiency and accuracy to cope with the complexity of feature selection for high-dimensional data more effectively. For instance, Hu et al. [25] introduced an enhanced black widow optimization algorithm aimed at feature selection, while Hammouri et al. [26] presented an improved dragonfly optimization approach for the same purpose. Similarly, Peng et al. [27] developed a feature selection method based on an optimized ant colony algorithm. Additionally, Li [28] proposed a local pair of bursary string gray wolf optimization algorithm for feature selection within the context of data classification. Al-Betar et al. [29] proposed an enhanced version of the electric eel optimization algorithm to solve the high dimensional feature selection problem, achieving efficient feature selection and classification accuracy. There are also many improved meta-heuristic algorithms used to improve the effectiveness of the feature selection problem including differential evolutionary algorithms [30], whale optimization [31], grey wolf optimization [32], ant colony optimization [33], and so on. Currently, there are also many researchers for processing medical data, for example, Braik et al. [34] proposed three binary white shark optimizer variants that significantly improve the classification accuracy and efficiency of feature selection in machine learning tasks by enhancing exploratory and exploitative mechanisms. Braik et al. [35] proposed a capuchin monkey search algorithm (CSA) in three adaptive versions (ECSA, PCSA, SCSA) to significantly improve the performance of feature selection (FS) in cognitive computing. Awadallah et al. [36] proposed six binary artificial rabbit optimization (ARO) variants to significantly improve the performance of feature selection (FS) for medical diagnostic data. Given the complex interwoven interactions among features in medical datasets, the large feature search space, and the stochastic nature inherent in existing technological tools, the task of feature selection still harbors great optimization potential. Given the complex and intertwined interactions among features in medical datasets, which leads to non-intuitive and unpredictable relationships among features; at the same time, medical datasets usually possess a large feature search space, which makes traditional feature selection methods face great computational challenges in processing [37]. In addition, existing technical means often carry a certain degree of randomness when performing feature selection, which not only affects the stability and reproducibility of the results, but also limits the optimization effect of the feature selection method. Therefore, despite the many advances that have been made in the field of feature selection, there still exists a great potential for optimization, especially in improving the accuracy, efficiency and stability of feature selection [38]. The current research difficulties lie in how to effectively cope with the complexity of medical datasets, as well as how to develop more robust and efficient feature selection methods to meet the urgent needs in practical applications [39]. To effectively address these challenges, the development of a comprehensive and advanced search strategy that can effectively circumvent local optimal solutions and robustly approximate the global optimal subset of features is of great significance in promoting research progress and achieving major breakthroughs in the field of feature selection for medical data.

Given this, this study deeply optimizes the red-billed blue magpie algorithm [40] to better meet the practical needs of feature selection for medical data, especially to cope with the complex interactions among features. Inspired by the survival strategy of red-billed blue magpies, this population-based optimization algorithm has gained significant attention across various fields. Its appeal stems from its ability to balance global search and local refinement, outstanding search performance, strong dynamic adaptability, and broad application potential. However, directly applying the red-billed blue magpie algorithm to the feature selection problem is challenging due to the complexity of feature interactions and the vast search space, which limit its ability to fully exploit its potential. To overcome this challenge, this study systematically optimizes the red-billed blue magpie algorithm for the special characteristics of the feature selection task while retaining its original efficient search capability. By introducing innovative behavioral mechanisms, the algorithm is endowed with a finer search strategy, enabling it to capture key feature combinations more acutely in the huge feature space. Meanwhile, the study focuses on addressing the trade-off between exploration and exploitation, leveraging the three core behavioral strategies of the red-billed blue magpie algorithm to enhance both global search and local optimization. And in the design process of IRBMO algorithm, we attach great importance to its simplicity and practicability, and do not rely on parameters, IRBMO algorithm is able to show excellent performance without the need of complex parameter tuning, and still show excellent performance. The key contributions of this paper are as follows:

(a) By simulating the natural behavior of the red-billed blue magpie, we propose a multi-behavioral improved IRBMO algorithm aimed at solving the medical data feature selection problem more effectively. The algorithm incorporates the following three key behavioral strategies:
  Ⅰ. Elite Search Behavior: Simulate the efficient guidance of red-billed blue magpie elite individuals to quickly locate the optimal feature combinations, combine random exploration and fine search strategy, and iteratively update to approach the global optimal feature solution.
  Ⅱ. Collaborative Hunting Behavior: Based on the concept of teamwork, the leader hunts for the optimal features together with the elites and random individuals, and flexibly adjusts the strategy to ensure that the optimal feature combinations are finally captured (i.e., found).
  Ⅲ. Memory Storage Behavior: Simulate the red-billed blue magpie’s strategy of storing food to cope with shortages, and when the current feature selection is insufficient, use the explored high-quality features to supplement them, so as to steadily advance the feature selection process and overcome the problem.
(b) To validate the performance of the IRBMO algorithm for feature selection of medical data, experiments were conducted in conjunction with a KNN classifier on 12 medical datasets of varying complexity.

The structure of the paper is as follows: Section 2 summarizes the literature review, Section 3 reviews the original RBMO algorithm, Section 4 introduces the IRBMO algorithm, Section 5 provides extensive experimental validation, and Section 6 concludes with discussions on future work.

## 2. Literature review

When dealing with diverse data, feature selection methods are favored by many scholars due to their high efficiency, among which, metaheuristic algorithms have been widely used in optimizing the feature selection problem, especially within the framework of packaged feature selection, which demonstrates significant advantages. It is well known that there is a wide variety of meta-heuristic algorithms for feature selection problems, covering group intelligence algorithms, evolutionary algorithms, algorithms based on physical principles, and innovative hybrid algorithms incorporating multiple strategies. The following is a carefully categorized overview of some of the metaheuristic algorithms applied to the feature selection problem.

Given the vast literature in the field of feature selection and the lack of uniform and standardized evaluation tools, it leads to the fact that different algorithmic strategies are often required for different datasets. Therefore, in this section the discussion focuses on the classification of feature selection algorithms based on meta-heuristic approaches.

### 2.1. Feature selection algorithms based on Swarm Intelligence

Swarm-based optimization algorithms have demonstrated a wide range of application potentials in the field of feature selection, successfully addressing the challenges of multi-dimensional and complex datasets. In 2021, Ma et al. [33] innovatively proposed a two-stage hybrid ant colony optimization algorithm (TSHFS-ACO) in response to the fact that the ant colony optimization algorithm, although possessing excellent search capabilities, is mainly limited to low-dimensional datasets. The algorithm accurately determines the size of the optimal feature subset by introducing an interval strategy, and guides the search process by combining feature relevance and classification performance, thus effectively alleviating the problem of search space explosion in high-dimensional feature selection. Experiments on several high-dimensional datasets show that TSHFS-ACO not only has excellent performance but also has a short runtime. In addition, Pan et al. [32] proposed an improved Gray Wolf optimization algorithm, which significantly improves the quality of the initial population in the feature selection task for high-dimensional data by fusing the ReliefF algorithm, Coupla entropy, competitive bootstrapping strategy, and Leader Wolf enhancement strategy based on differential evolution, and enhances the search flexibility and global search capability of the algorithm. This improvement effectively avoids the problem of local optimal solution, and experimental results on 10 high-dimensional small-sample gene expression datasets show that the algorithm is able to achieve a significant improvement in classification accuracy with a very low feature selection ratio (less than 0.67%), which is remarkably competitive with existing state-of-the-art feature selection methods. The comprehensive study shows that the algorithm achieves a good balance between exploration and exploitation, and its unique search strategy significantly improves the search performance of the Gray Wolf optimization algorithm. In addition, Braik et al. [34] conducted an in-depth study on the application of White Shark Optimizer (WSO) in the field of feature selection and designed three innovative binary variants - Binary Adaptive WSO (BAWSO), Binary Comprehensive Learning WSO (BCLWSO) and Binary Heterogeneous CLWSO (BHCLWSO). These variants effectively balance the exploration and exploitation mechanisms and address the problems of low solution accuracy and slow convergence speed faced by WSO in optimization tasks. Comprehensive evaluation results on 24 well-known datasets show that these variants significantly improve the performance of classical binary WSO (BWSO), especially BHCLWSO, which demonstrates superiority over other binary algorithms in terms of classification accuracy and number of feature selections. Nadimi-Shahraki et al. [41] proposed an Aquila Optimizer (AO)-based wrapper feature selection method for addressing the negative impact of redundant and irrelevant features on algorithm performance in medical datasets. By introducing the S-shaped Binary Aquila Optimizer (SBAO) and V-shaped Binary Aquila Optimizer (VBAO), the method is able to efficiently filter out the optimal feature subset and significantly improve the accuracy of the classification task. Experiments on seven benchmark medical datasets show that SBAO and VBAO outperform six state-of-the-art binary optimization algorithms in classification performance. In addition, tests on the COVID-19 real dataset further validate the superiority of SBAO in terms of the number of feature selections and classification accuracy. Nadimi-Shahraki et al. [42] proposed an Improved Grey Wolf Optimizer (I-GWO) aimed at solving the problems of insufficient population diversity, exploitation and exploration imbalance, and premature convergence, which are common in global optimization and engineering design problems. The algorithm introduces a dimensional learning-based hunting (DLH) strategy that enhances the balance between local and global search and maintains population diversity by modeling individual wolf hunting behavior and constructing unique neighborhoods for each wolf to share information. Experiments on the CEC 2018 benchmark suite and four engineering design problems show that I-GWO outperforms six state-of-the-art meta-heuristic algorithms in terms of performance, and exhibits significant competitiveness and efficiency especially in engineering design problems. Nadimi-Shahraki et al. [43] proposed a discrete version of the Improved Grey Wolf Optimizer (I-GWO), known as DI-GWOCD, for the problem of community detection in complex networks.The algorithm optimizes node allocation using the local search strategy of I-GWO and introduces the Binary Distance Vector (BDV) to solve the discrete community detection problem. Evaluated by experiments on several real-world network datasets, DI-GWOCD excels in metrics such as modularity and NMI. Compared with existing algorithms, DI-GWOCD is able to detect communities with higher quality, demonstrating its advantages in large-scale network community detection.

### 2.2. Evolutionary-based feature selection algorithms

In order to improve the performance of feature selection problems and simultaneously reduce the number of features and computational burden, evolutionary algorithms have achieved significant development and innovation in recent years. Among them, classical algorithms such as genetic algorithm [44] and differential evolution [45] are widely used to solve various challenges in feature selection. To address the challenges of feature selection for high-dimensional data, Ma et al. [46] proposed a novel roulette-based level learning evolutionary algorithm (RWLLEA) in this study. The algorithm enhances the diversity of the population by introducing a balanced population model and incorporates a dynamic search space update strategy to effectively reduce the computational cost. Experimental validation results show that RWLLEA is able to obtain a streamlined feature set with shorter runtime on 15 different datasets while exhibiting superior classification accuracy, which significantly outperforms the other six feature selection techniques. In addition, Espinosa et al. [47] innovated in agent-assisted multi-objective evolutionary algorithms by skillfully fusing generation-fixed evolutionary control with a direct fitness replacement strategy. By continuously updating the LSTM agent model through an incremental learning mechanism, the method effectively accelerates the process of time series feature selection. In tasks such as air quality prediction, indoor temperature of smart buildings, and oil temperature prediction of power transformers, the algorithm achieves a significant improvement in prediction performance compared to traditional and existing agent-assisted feature selection methods, by 23.98%, 34.61%, and 13.77%, respectively. These results not only demonstrate the strong potential of evolutionary algorithms in the field of feature selection, but also provide new ideas and methods for future research. Nadimi-Shahraki et al. [48] proposed a Multi-Trial Vector based Differential Evolutionary Algorithm (MTDE), which significantly improves the performance of the algorithm through the introduction of an adaptive moving step and the Multi-Trial Vector Method (MTV). The MTV method combines three kinds of trial vector producers (TVPs): representative-based, locally randomized, and global best-history-based TVPs, and achieves experience sharing through a winner distribution strategy and a lifecycle archiving mechanism. Experiments on the CEC 2018 benchmark suite show that MTDE exhibits higher accuracy and performance in dealing with problems of different complexity, outperforming state-of-the-art meta-heuristic algorithms such as GWO, WOA, SSA, HHO, etc.

### 2.3. Physics-based feature selection algorithms

Many feature selection algorithms are inspired by physical phenomena.

Houssein et al. [49] addressed the challenge of high-dimensional feature selection in liver disease datasets by pioneering the enhanced Keplerian optimization algorithm, I-KOA. This algorithm ingeniously incorporates the local escape operator derived from dyadic learning and the k-nearest-neighbor classifier. Following a series of rigorous experimental validations, I-KOA demonstrated exceptional performance on the complex liver disease dataset. It surpassed multiple optimization algorithms in various aspects, including classification accuracy, number of selected features, sensitivity, precision, and F1 scores. Furthermore, I-KOA provides a highly efficient and precise feature selection tool for medical diagnosis decision support systems, showcasing significant practical application value and profound implications. Meanwhile, Abdel-Salam et al. [50] proposed the ACRIME algorithm to address the shortcomings of the RIME optimization algorithm in terms of the balance between exploration and exploitation, the avoidance of local optimal solutions, and the convergence speed, etc. ACRIME is effective in enhancing the population diversity and optimizing the population by introducing four innovative strategies, such as the initialization of chaotic mapping, the co-integration phase of the search for self-adaptive symbiotic organisms (SOS), the hybrid mutation strategy, and the restart strategy. enhanced population diversity, optimized the balance between exploration and exploitation, and substantially improved local and global search capabilities. The experimental results show that ACRIME performs excellently in CEC2005 and CEC2019 benchmark function tests, and also demonstrates strong competitiveness in feature selection applications on 14 datasets and COVID-19 classification real-world problems, and its performance is significantly better than that of other classical and advanced meta-heuristic algorithms. In addition, Zhang et al. [51] proposed a plant root growth optimization (PRGO) algorithm by drawing inspiration from nature and inspired by the plant rhizome growth mechanism. The algorithm skillfully combines the global exploration and local exploitation search strategies, and demonstrates excellent performance on both CEC2014 and CEC2017 test sets. For the high-dimensional feature selection problem, the binary variant of PRGO, BPRGO, is comprehensively compared with eight well-known methods on 16 datasets, and demonstrates its stronger feature reduction ability and better overall performance through a number of performance metrics. BPRGO is capable of obtaining a very small feature subset while maintaining high accuracy, providing a novel and effective solution to the high-dimensional feature selection problem.

### 2.4. Human-based feature selection algorithms

With the development of data processing, many human-based feature selection methods have been proposed.

Zhuang et al. [52] proposed Binary Arithmetic Optimization Algorithm (BAOA), which enhances exploration and exploitation by redesigning the multiplication operator and introducing four families of transfer functions. Further, a parallel mechanism is introduced to form a parallel binary AOA (PBAOA). Based on this, a four-family transfer function is introduced to enhance the performance of the algorithm. To further enhance the efficiency, this paper incorporates the parallel mechanism into BAOA and proposes the Parallel Binary AOA (PBAOA) algorithm. Tested on 10 low-dimensional and 10 high-dimensional datasets from UCI and scikit feature libraries, the results show that BAOA and PBAOA outperform the classical and the latest algorithms, and the transfer function selection varies according to the dimensionality of the dataset, and the parallel mechanism is effective in enhancing the performance of the algorithm. Meanwhile, Cinar. [53] proposed the adaptive modulo binary optimization (AMBO) algorithm for feature selection to effectively deal with the NP-Hard problem and dimensionality catastrophe. AMBO adopts a binary optimization strategy combined with adaptive mutation and crossover mechanisms, and innovatively introduces binary logic gates modulo intelligent local search. Experiments show that AMBO performs well on 11 datasets when comparing algorithms such as BPSO and multiple genetic algorithms, and statistical tests confirm its significance. When compared to other metaheuristic algorithms on 21 datasets, AMBO outperforms in terms of classification error rate, fitness, and feature selection, demonstrating superior performance. In addition, Khosravi et al. [54] proposed a novel binary group teaching optimization algorithm, BGTOALC, which combines local search with chaotic mapping and aims at solving the high-dimensional feature selection problem. BGTOALC enhances the exploration and exploitation of the algorithm by introducing the novel binary operators of Binary Teacher Phase Good Group (BTPGG) and Binary Teacher Phase Bad Group (BTPBG). Meanwhile, the student phase uses the new binary student’s objection-based learning (BSOBL) operator to enhance the performance of the algorithm by utilizing the objection strategy. In addition, the algorithm is designed with the Mean Binary Selection (MBS) operator to optimize the teacher assignment phase in a binary manner to improve the convergence rate. For further comparison, the study also developed the BGTOAV and BGTOAS algorithms using S-shaped and V-shaped transfer functions. Experimental results show that the BGTOALC method outperforms the previous methods in reducing the number of features and improving the accuracy of the machine learning algorithms on a dataset of 30 different dimensions. Statistical analysis further confirms that BGTOALC outperforms other binary metaheuristic algorithms in terms of efficiency and convergence rate. Nadimi-Shahraki et al. [55] proposed an improved algorithm based on multiple test vectors (MTV-SCA) to address the problems of the sinusoidal cosine algorithm (SCA), which is prone to fall into the local optimum, the exploration-exploitation imbalance, and the lack of accuracy. MTV-SCA effectively balances exploration and exploitation and avoids premature convergence by introducing the multi-test vector (MTV) method and combining four different search strategies. Experiments on CEC 2018 benchmark functions show that MTV-SCA outperforms conventional SCA and other advanced algorithms (e.g., CEC 2017 winner algorithm) in terms of convergence speed, accuracy, and avoidance of local optima. Statistical tests (Friedman and Wilcoxon signed rank test) further validate its significant advantages. In addition, the successful application of MTV-SCA to six non-convex constrained engineering design problems demonstrates its practical applicability in complex optimization tasks.

### 2.5. Hybrid filter-wrapper feature selection models

When dealing with high-dimensional datasets, feature selection is a key step to improve the effectiveness of machine learning methods. Currently, the mainstream solutions to the FS problem fall into two main categories: packing and filtering methods. In order to optimize the performance of the FS task, a strategy that combines these two methods is often used in the literature [56]. This combination is usually divided into two phases: first, the most critical features are filtered out using the filter approach; then, in the second phase, the optimal subset of features is further identified from these features by the wrapper approach.

Wrapper and filter feature selection are widely used when dealing with the challenges of high-dimensional data. Often, a hybrid of these two approaches is used. Vommi et al. [57] proposed a hybrid feature selection approach for medical dataset classification challenges, combining ReliefF and fuzzy entropy (RFE) filtering techniques and incorporating Enhanced Equilibrium Optimizer (EO) techniques including Opposition-Based Learning (OBL), Cauchy Variation Operator, and Novel Search Strategies. The enhanced EO is integrated with eight time-varying S- and V-shaped transfer functions to form a binary-enhanced equilibrium optimizer (BEE) for extracting essential features from the integrated features. Experiments are validated on 22 benchmark datasets and four microarray datasets (including the COVID-19 case), and the results show that the RFE-BEE method outperforms other state-of-the-art algorithms in terms of fitness, accuracy, precision, sensitivity, and F-measure. Song et al. [58] proposed a hybrid feature selection method, SFEMEO, based on an elite-guided mutation strategy, designed for high-dimensional, multi-sample and multi-categorical cancer gene expression data to solve the cancer subtype diagnosis problem. The method is divided into two phases, initial screening and combinatorial modeling, using seven filtering methods combined with logical operations to generate feature subsets and thresholds determined by leave-one-out cross-validation. On the UCI dataset, SFEMEO improves the classification accuracy by 0.56% to 20.19% compared to the other nine algorithms, and the optimal fitness is significantly improved. On the cancer gene expression dataset, SFEMEO improves the accuracy by 3.73% to 18.13% with superior best fit performance compared to the 9 intelligent optimization algorithms. The Wilcoxon rank sum test validates the effectiveness of SFEMEO and its advantages in solving the feature selection problem for high-dimensional cancer gene expression data.

## 3. Review of RBMO

### 3.1. Inspiration for RBMO

The red-billed blue magpie, a unique Asian bird treasure, has a wide distribution range covering China, India and Myanmar. They are known for their robust physique, dazzling blue plumage and bright red bill, adding a splash of color to nature. The red-billed blue magpie exhibits significant dietary diversity, consuming insects, small vertebrates, and various plant fruits. Their feeding strategies are flexible and include walking on the ground, jumping and weaving between branches, especially in the early morning and at dusk. Typically, red-billed blue magpies travel in small groups of two to five individuals, occasionally forming larger flocks with pronounced social behaviors. Their social interactions are especially prominent when hunting cooperatively. Once they find food, such as fruit or insects, they quickly signal to gather their companions and work together to round up the target. This tacit teamwork allows them to easily break through their prey’s defenses. In addition, red-billed blue magpies also show unique storage behavior, hiding their food in hidden places such as tree holes, gaps between branches or rock crevices to avoid being snatched by other predators.

Overall, red-billed blue magpies are not only highly skilled foragers, but also excel at diversifying their food reserves and display excellent teamwork and social intelligence in hunting. It is these unique ecological characteristics that provide a rich source of inspiration for the design of the RBMO metaheuristic algorithm.

### 3.2. Mathematical modeling of RBMO

#### 3.2.1. Initialization stage.

The population of red-billed blue magpies is first created randomly, i.e., assuming that N red-billed blue magpies are in a D-dimensional space, with each red-billed blue magpie located:


$$\begin{array}{c} \begin{array}{c} X=[\begin{array}{cc} \begin{array}{ccc} x_{1,1} & x_{1,2} & \cdots \\ x_{2,1} & x_{2,2} & \cdots \\ \vdots & \vdots & \ddots \end{array} & \begin{array}{ccc} x_{1,j} & \cdots & x_{1,D}\\ x_{2,j} & \cdots & x_{2,D}\\ \vdots & \ddots & \vdots \end{array}\\ \begin{array}{ccc} x_{i,1} & x_{i,2} & \cdots \\ \vdots & \vdots & \ddots \\ x_{N,1} & x_{N,2} & \cdots \end{array} & \begin{array}{ccc} x_{i,j} & \cdots & x_{i,D}\\ \vdots & \ddots & \vdots \\ x_{N,j} & \cdots & x_{N,D} \end{array} \end{array}]N_{\times D\;} \end{array} \end{array}$$ (1)

where *i* = 1,2,...,*N*, *j* = 1,2,...,*D*, $X_{i}^{j}$denotes the position of the *ith* red-billed blue magpie in dimension *j*. *N* is the population size of red-billed blue magpies and *D* is the dimension of the problem.

#### 3.2.2. Search behavior.

Red-billed blue magpies typically forage in small groups, which facilitates a more efficient search and better population survival. They search for food in the forest or on the ground by hopping and flying. This adaptability enables red-billed blue magpies to adopt various hunting strategies based on environmental factors and resource availability, ensuring a steady food supply. When groups gather to forage, the mathematical model is as follows:


$$\begin{array}{c} \begin{array}{c} X_{i+1}=X_{i}+rand1\times \left(\frac{1}{n}\times \sum_{k}^{n}X_{k}-X_{rs}\right) \end{array} \end{array}$$ (2)

where *X*_*i*_ represents the *i-th* individual, *n* denotes the number of groups of red-billed blue magpies randomly chosen from the search population, ranging from 2 to 5, *X*_*k*_ refers to the *k-th* randomly selected individual, and *X*_*r*_ represents the search agent chosen at random during the current iteration.


$$\begin{array}{c} \begin{array}{c} X_{i+1}=X_{i}+rand2\times \left(\frac{1}{m}\times \sum_{k}^{m}X_{k}-X_{rs}\right) \end{array} \end{array}$$ (3)

where *m* represents the number of search agents in the cluster exploring for food, ranging from 10 to N. The number of search agents is selected randomly from the entire population.

#### 3.2.3. Preying behavior.

Red-billed blue magpies demonstrate remarkable hunting abilities and teamwork, particularly when hunting prey. They employ a range of tactics, including rapid pecking, jumping, and aerial capture of insects. Individually or in small groups, they tend to hunt small prey and gather plants, a process that can be mathematically described by Equation (4). When grouped together, red-billed blue magpies are able to attack larger targets such as large insects and even small vertebrates in a concerted manner, and this collective hunting behavior is mathematically modeled in Equation (5). This range of predatory strategies highlights the diverse talents and high adaptability of red-billed blue magpies as predators, ensuring that they are able to forage efficiently in different environments.


$$\begin{array}{c} \begin{array}{c} X_{i+1}=X_{food}+CF\times rand1\times \left(\frac{1}{n}\times \sum_{k}^{n}X_{k}-X_{rs}\right) \end{array} \end{array}$$ (4)

$$\begin{array}{c} \begin{array}{c} X_{i+1}=X_{food}+CF\times rand2\times \left(\frac{1}{m}\times \sum_{k}^{m}X_{k}-X_{rs}\right) \end{array} \end{array}$$ (5)

where $X_{food}$ represents the location of the food, $CF={\left(1-(t/T)\right)}^{\left(2t/T\right)}$, is the conditioning factor, and *rand* denotes a value randomly chosen from the range [0, 1].

#### 3.2.4. Storage behavior.

When red-billed blue magpies hunt a surplus of food, they store it in a tree hole or hidden place for the winter food shortage season. This process can be formulated as preserving the optimal solution to more effectively identify the global optimum. The corresponding mathematical model is:


$$\begin{array}{c} \begin{array}{c} X_{i+1}=\left\{ \begin{array}{c} {\;X}_{i}\;if\;{fitness}_{old}>{fitness}_{new}\\ \;X_{i+1}\;else\; \end{array}\right.\;\# \end{array} \end{array}$$ (6)

where *fitness*_*old*_ and *fitness*_*new*_ denote the fitness values of the red-billed blue magpie following position updates, respectively.

In the RBMO algorithmic framework, the optimization process starts with the construction of a randomly generated set of candidate solutions, which is imaginatively referred to as a “population”.The core strategy of RBMO revolves around cyclic paths, and the goal is to find the optimal or near-optimal solution. A distinct “food reserve” mechanism improves the algorithm’s ability to explore and exploit the solution space, enabling more efficient search. The search continues until a specified termination condition is satisfied. The pseudo-code for RBMO is provided in Table 1.

**Table 1:** Pseudo Code of RBMO.

Algorithm 1 Pseudo Code of RBMO
1: Initialize Problem Setting (D, lb, ub, N, $T_{\max}$, t) 2: Initialize the solution positions randomly 3: Calculate the fitness function 4: Find the best position 5: **While** $t<T_{\max}$ **do** 6: **For** *i* <N 7: Search Behavior 8: if rand<p 9: Using Equation (2) 10: else 11: Using Equation (3) 9: Predatory behavior 13: if rand<p 14: Using Equation (4) 15: else 16: Using Equation (5) 17: Storage Behavior 18: Using Equation (6) 19: **end** 20: Update the optimal Red-billed blue magpie 21: **end** 22: Return best solution

## 4. Improved version of the RBMO algorithm

### 4.1. Motivation

The RBMO (Red-billed Blue Magpie Optimization) algorithm is inspired by the red-billed blue magpie, an efficient predator in nature, whose flexible and varied foraging strategies and highly collaborative group behaviors provide important inspirations for the algorithm design. The RBMO algorithm offers distinct advantages in both exploration and exploitation of the search space. However, its application to the complex, high-dimensional task of feature selection in medical data still presents several challenges. The difficulties in the field of medical feature selection mainly include the huge search space, the complex and nonlinear relationship between high-dimensional features, and the bottleneck of computational efficiency. To overcome these challenges, this paper proposes an enhanced version of IRBMO algorithm based on an in-depth analysis of the characteristics of the RBMO algorithm and customized improvement of the original algorithm with the actual needs of medical data feature selection.

Within the framework of an improved optimization algorithm for red-billed blue magpies, we innovatively propose an enhanced set of behavioral strategies designed to significantly improve the performance and efficiency of the algorithm in the task of feature selection for medical data. These well-designed strategies specifically include elite search behavior, collaborative hunting behavior, and memory storage behavior, each of which is targeted to address the complexity and high dimensionality challenges in medical data processing. First, elite search behavior is introduced to mimic the efficient exploration patterns of good individuals (i.e., elites) in nature. By retaining and reinforcing those solutions (feature subsets) that perform well during the algorithm iteration process, the elite search behavior is able to guide the search process to quickly converge to a global or local optimal solution, thus quickly identifying the most representative features in a huge medical dataset.

Second, collaborative hunting behavior emphasizes information sharing and cooperative work among individuals within a population. This strategy mimics the close collaboration of red-billed blue magpie groups during hunting, boosting the algorithm’s global search efficiency and solution diversity by promoting the exchange and integration of information among different solutions. In the context of feature selection for medical data, this means that the algorithm is able to explore the feature space more efficiently, discovering combinations of features that may be overlooked when considered individually but that have significant predictive value when combined. Finally, the memory storage behavior is a manifestation of a mechanism for long-term learning and knowledge accumulation. The strategy works by creating a memory store in the algorithm for storing excellent solutions and their related information encountered in historical iterations. This not only helps to avoid the algorithm from falling into the predicament of repeated searches, but also speeds up the optimization process by reusing this valuable information in subsequent iterations. For medical data feature selection, the memory storage behavior ensures that the algorithm can quickly leverage previous experience when faced with similar or related datasets, improving the accuracy and efficiency of feature selection.

The IRBMO algorithm not only retains the core advantages of RBMO in search and optimization, but also incorporates a series of innovative mechanisms by drawing on the survival wisdom of the red-billed blue magpie in its natural environment. These enhancements allow IRBMO to better address the challenges of high-dimensional feature spaces, accurately capture complex nonlinear relationships between features, and preserve computational efficiency when processing large-scale datasets. In the medical feature selection task, IRBMO demonstrates excellent performance, providing an efficient and reliable solution for the field.

### 4.2. Elite search behavior

**Fig 1:**
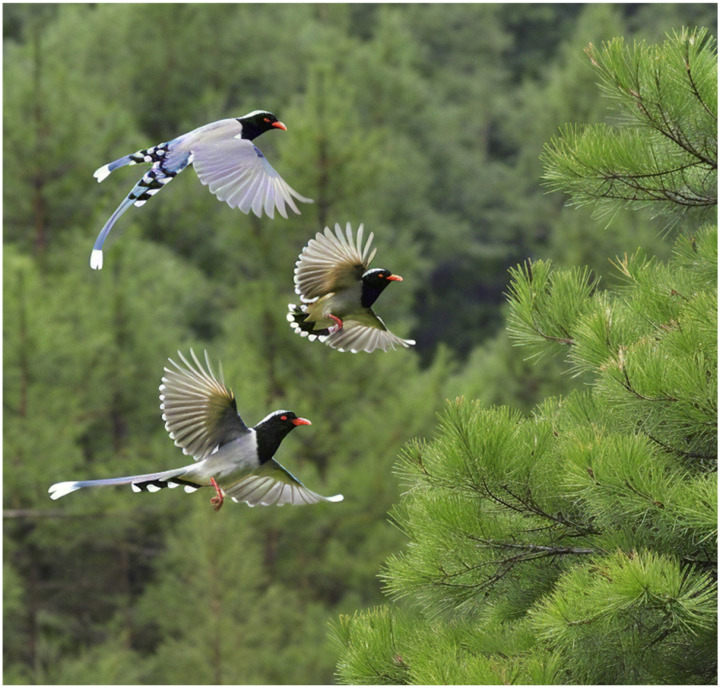
Elite search behavior.

The search behavior of red-billed blue magpies is a team action and very efficient, in the algorithm simulation, we analogize its elite search behavior to the process of feature selection optimization to quickly lock the optimal features. The elite search behavior of the red-billed blue magpie is shown in Fig 1.

Firstly, the algorithm is guided by the elite individuals of red-billed blue magpies, selecting the three individuals with the highest fitness (elite individuals) and calculating the average value of their positions to guide the search process towards the region of high-quality feature solutions, and then switching between the exploration based on the average positions of the elite individuals and the fine search based on the randomly selected elite individuals and the principle of Brownian Motion, and then, in the stage of fine search, the algorithm introduced the Brownian Motion random vectors to increase the diversity and flexibility of the search direction, and finally gradually approaches the optimal feature solution by updating the position of each search individual through efficient iteration. Its mathematical model is as follows:


$$\mathrm{\backslash [}\begin{array}{c} X_{i+1}=X_{1}+rand\times \left(X_{eq}-X_{R1}\right)\# \end{array}\mathrm{\backslash ]}$$ (7)

where $X_{1}$ represents the current optimal individual and $X_{eq}$ represents the average of the top three current optimal individuals. The position of an individual moves towards a random point between its current position and the average position of the three optimal individuals ($X_{eq}$). This movement simulates the exploration behavior of moving closer to the optimal feature solution.


$$\mathrm{\backslash [}\begin{array}{c} X_{i+1}=RS+rb\times rand\times \left(X_{best}-X_{R1}\right)\# \end{array}\mathrm{\backslash ]}$$ (8)

where *RS* represents the elite pool, i.e., the optimal three individuals and their average values are placed in it, from which one is drawn each time, $X_{best}$ represents the current optimal individual, and *rb* is the Brownian motion adjustment factor. That is, one is randomly selected from the optimal three individuals and their average positions, and then the positions of the individuals are updated according to the principle of Brownian motion, which simulates a more random exploration process.

### 4.3. Collaborative hunting behavior

**Fig 2:**
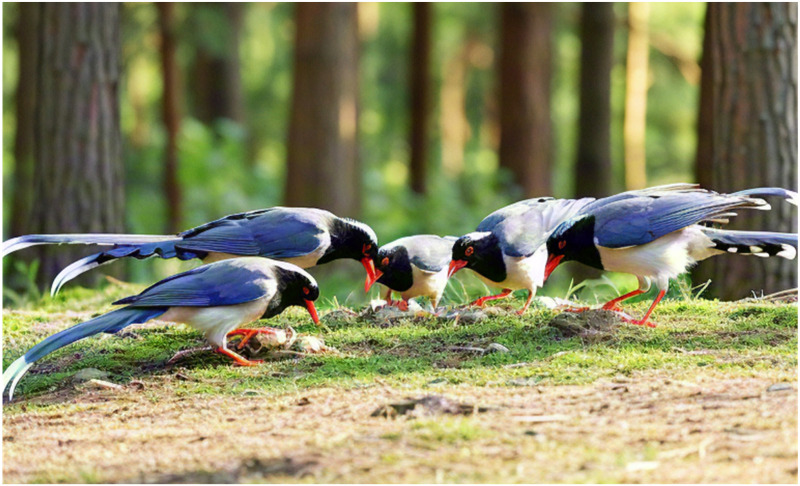
Collaborative hunting behavior.

When red-billed blue magpies find prey, they will engage in teamwork to capture prey, based on this, we redesigned a behavioral mathematical model based on collaborative hunting. Collaborative hunting behavior is shown in Fig 2.

Firstly, based on the idea of teamwork of red-billed blue magpies, the prey is constantly surrounded by the team leader (the optimal individual) with the elites in the elite pool as well as the random individuals, and then the position between the elites and the random group is constantly adjusted through the Cauchy distribution to encircle the prey. If the prey escapes from the encirclement, then it is up to the leader and the elites to re-encircle the escaped prey and adjust the encirclement between the leader and the elites through levy flights. The mathematical model is as follows:


$$\mathrm{\backslash [}\begin{array}{c} X_{i+1}=X_{best}+cv\times \left(X_{eq}-X_{i}\right)\# \end{array}\mathrm{\backslash ]}$$ (9)

where *cv* represents the Cauchy distribution and *L* denotes the moderator.


$$\begin{array}{c} \begin{array}{c} X_{i+1}=\left(RS-X_{best}\right)\times levy+rand\times RS\times L \end{array} \end{array}$$ (10)

### 4.4. Memory storage behavior

**Fig 3:**
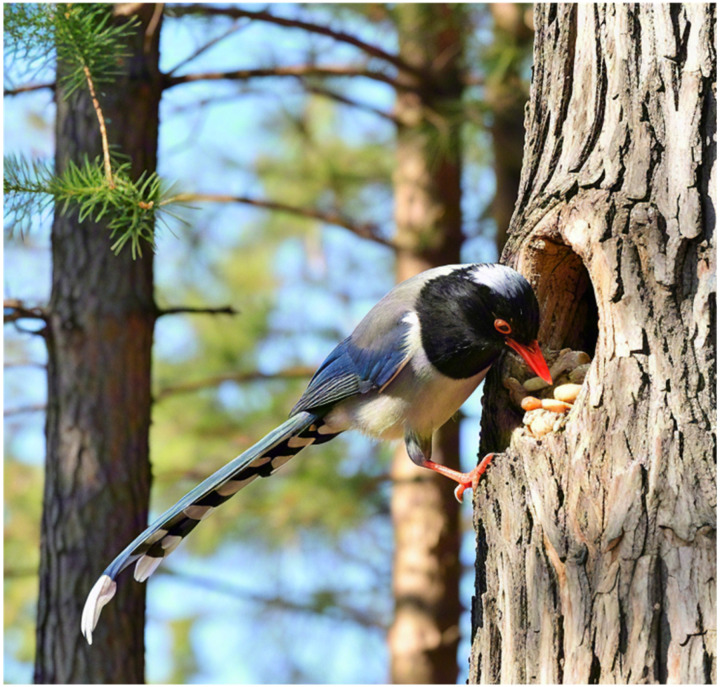
Memory storage behavior.

To survive cold winters when food is scarce, red-billed blue magpies have evolved the behavior of storing food, thus ensuring a stable food supply in times of shortage. The memory storage behavior is shown in Fig 3.

If they find that the food they hunt cannot supply the entire population, they begin to adaptively compensate by consuming stored food to survive the winter. Their mathematical model is as follows:


$$\mathrm{\backslash [}\begin{array}{c} X_{i+1}=\left\{ \begin{array}{c} X_{i}\;if\;{fit}_{old}>{fit}_{new}\\ {\;X}_{i+1}\;else\; \end{array}\right.\;\# \end{array}\mathrm{\backslash ]}$$ (11)

where if *fit*_*old*_ and *fit*_*new*_ denote the fitness and current fitness in the memory respectively. The decision to update the memory is made by comparing the current fitness value with the fitness value in the memory.


$$\mathrm{\backslash [}\begin{array}{c} \left\{ \begin{array}{c} {\;X}_{i+1}=X_{i}+CF\times \left(RS+rand\times (ub-lb)\right)\times E\;if\;A>rand\;\\ \;X_{i+1}=X_{i}+\left(A\times (1-rand)+rand\right)\times \left(X_{idx1}-X_{idx2}\right)\;else\; \end{array}\right.\;\# \end{array}\mathrm{\backslash ]}$$ (12)

where *A* is the determinant, if food is abundant then the elite individuals continue to lead the group in hunting, if food is scarce then the group uses the reserves for group survival. *X*_*idx*1_ and *X*_*idx*2_ are randomly selected individuals in the group. Therefore, the pseudo-code of IRBMO is shown in Table 2:

**Table 2:** The Pseudo-Code of the IRBMO Algorithm.

**Algorithm 2:** Pseudo-code of the IRBMO algorithm
1：Initialize the IRBMO parameters 2：Calculate the fitness function 3：Find the best position 4：Boundary check and correction 5：Start searching function 6： **While** t < 1 + T 7： **For** i = 1 to N do 8： Behavior 1: Elite search behavior 9： **IF** rand < S 10： Update position using Equation (7) 11： **Else** 12： Update position using Equation (8) 13： **End** 14： Behavior 2: Collaborative hunting behavior 15： **IF** rand<S 16： Update position using Equation (9) 17： **Else** S = 3 18： Update position using Equation (10) 19： **End** 18： Behavior 3: Memory storage behavior 19： Update position using Equation (11) 20： Update position using Equation (12) 21： **End** 22： Update the optimal Red-billed blue magpie 23： **End** 24：Return the best solution 25：**End**

### 4.5. Space-time complexity

The time and space complexity of the RBMO algorithm can be summarized as follows:

Regarding time complexity, the RBMO algorithm is primarily influenced by the maximum number of iterations *T*, the population size *N*, and the dimensionality of the individuals (*dim*). Each iteration involves steps such as individual fitness calculation, position update, boundary condition processing, etc., and the time complexity of these steps is directly related to *N* and *dim*. Additionally, the time complexity of the sorting step reaches O(N log N). The overall time complexity can be simplified to O(T * N * (dim + log N)) when secondary factors such as the computation of fitness function are ignored, which intuitively shows that the algorithm’s time consumption increases notably with the number of iterations, population size, and individual dimensionality.

In terms of space complexity, the main space occupation of the RBMO algorithm stems from the population matrix *X*, which has a space complexity of O(N * dim) and is responsible for storing the positional information of *N* individuals in the dimensional space of dim. Although the algorithm also needs to store auxiliary data such as fitness vectors, historical locations, indexes, etc., the space complexity of these data is relatively low and has a negligible effect on the total space complexity. Therefore, the total space complexity is mainly determined by the population matrix *X*, i.e., O(N * dim), which clearly indicates that the algorithm’s space requirement is directly related to both the population size and individual dimensions.

### 4.6. The binary IRBMO

To overcome the limitations of the meta-heuristic algorithm in dealing with the binary problem of feature selection, we transform it to adapt the algorithm from the continuous search space to the binary domain. Taghian et al. [59] summarized the application of transfer function based binary meta-heuristic algorithms in feature selection, where various binarization techniques are summarized and classified into four main categories, i.e., normalization, angular modulation, binary operators and transfer functions. Normalization normalizes the solution in a continuous space to the range [0, 1] and then converts it to a binary value by thresholding (e.g., 0.5). Angular modulation is the use of trigonometric functions (e.g., sine and cosine) to map real solutions to binary arrays. Normalization and angle modulation are simpler methods of binary conversion for specific problem scenarios. Binary Operators use binary operators (e.g., XOR, AND, OR, NOT) to generate new binary solutions, operate directly in binary space, and are suitable for discrete optimization problems, and Transfer Functions use transfer functions to map real solutions to binary values. The transfer function calculates the probability of a change in the value of each dimension and is the most commonly used method that preserves the search properties of continuous algorithms and is suitable for a wide range of optimization problems.

In this research, we realize the mapping of continuous solutions to binary representations by designing a transformation function (TF) that allows the algorithm to operate effectively in feature selection problems. Specifically, the TF determines the status of each feature by means of a threshold decision based on the importance score of the feature: if the score is higher than a predefined threshold, it is labeled as “1” (selected); otherwise, it is labeled as “0” (excluded). This approach retains the powerful global search capability of the metaheuristic algorithm, while simplifying the feature selection process and providing new possibilities for its application to discrete optimization problems. The transformation logic is briefly described as follows:


$$\begin{array}{c} \begin{array}{c} {{(X}_{i})}_{b}=\left\{ \begin{array}{c} 1,{\;X}_{i}>0.5\\ 0,{\;X}_{i}<0.5 \end{array}\right. \end{array} \end{array}$$ (13)

In the subsequent experimental chapters, we also further explore the transformation effects of the S- and V-shaped transfer functions to verify their effectiveness in the feature selection task.

## 5. Experimental results and discussion

In order to comprehensively and thoroughly measure the efficacy of our proposed binary version of IRBMO in real-world applications, we have carefully planned and executed a series of extensive and systematic experimental validations. These experiments are constructed based on a diverse dataset of 12 real medical scenarios, each of which varies in the number of features, sample size, and category distribution to ensure the broad representativeness and practicality of the experimental results. In terms of experimental design, we adopted a diverse comparison strategy, pitting the IRBMO algorithm against a variety of meta-heuristic algorithms with their own unique characteristics. This approach enables us to analyze the performance characteristics of IRBMO, including its strengths and limitations, in a nuanced manner, which lays a solid foundation for subsequent algorithm iterations and performance enhancement. In addition, we also evaluated our algorithm against the current mainstream feature selection algorithms, aiming to not only validate its competitiveness, but also explore the unique advantages of the algorithm in specific application scenarios through this session. Further, in order to explore the optimal classification synergy, we implemented an experiment combining the IRBMO algorithm with the KNN [60] classifier, aiming to further enhance the accuracy and efficiency of the classification task through the strong combination of the two. And we also combine IRBMO with transfer functions to construct variants with stronger generalization capabilities. The design and implementation of this series of comprehensive experiments not only improved our overall insight into the algorithm’s performance, but also provided a strong support for the efficient application of the algorithm in real-world problems.

### 5.1. Description of the datasets

To evaluate and validate the effectiveness of the new algorithm in this study in a comprehensive and detailed manner, we carefully selected 12 unique datasets covering multiple fields of medicine from several well-known data warehouses such as UCI and KEEL. These datasets include Breast Cancer Coimbra, Wisconsin Diagnostic & Prognostic Breast Cancer, Cleveland Heart Disease, Dermatology, Diabetic Retinopathy Debrecen, Hepatitis, ILPD, Lymphography, Parkinson’s Disease (both generic and classification datasets), and Spectfheart. These selected datasets have distinctive characteristics, ranging from 79 to 1151 sample sizes and feature dimensions from 9 to 753, demonstrating the adaptability and effectiveness of the algorithms in addressing the challenges of data of different sizes and complexities. Detailed dataset information is summarized in Table 3, where the abbreviated form of the dataset is used to enhance readability, and core parameters such as data source, sample size, number of features, number of categories, and the research area to which it belongs are fully documented. In particular, it is noted that most of the selected datasets focus on binary classification problems, which helps to accurately assess the performance of the algorithms in such tasks; meanwhile, the existence of a few multicategorical datasets provides a valuable opportunity to validate the algorithms in terms of their ability to recognize multiple categories. To ensure the comprehensiveness and rigor of the algorithm’s performance validation, we follow the standard dataset partitioning principle and allocate 75% of each dataset for training and the remaining 25% for testing. This division strategy aims to balance the sufficient sample size required for algorithm learning with the effective evaluation of the algorithm’s generalization ability in the test set, so as to ensure the accuracy and reliability of the algorithm’s performance evaluation results.

**Table 3:** Description of the datasets.

Dataset	Source	No. of Samples	No. of Features	No. of Classes	Domain
BCC	UCI	116	9	2	Medicine
BCWD	UCI	569	30	2	Medicine
BCWP	UCI	198	33	2	Medicine
Cleveland	KEEL	297	13	5	Medicine
Dermatology	UCI	366	34	6	Medicine
DRD	UCI	1151	19	2	Medicine
Hepatitis	KEEL	79	18	2	Medicine
ILPD	UCI	583	10	2	Medicine
Lymphography	UCI	148	18	4	Medicine
Parkinsons	UCI	197	22	2	Medicine
PDC	UCI	756	753	2	Medicine
Spectfheart	KEEL	266	44	2	Medicine

### 5.2. Performance measures

In this research, the effectiveness and accuracy of the proposed algorithm is validated against other comparative algorithms by using various performance metrics. These performance metrics are specifically defined as shown in the following section.

#### 5.2.1. Evaluation metrics.

1. Average fitness value: This performance metric is used to measure the quality of the selected subset of features in the target solution. The formula is shown in the mathematical expression below:

$$\mathrm{\backslash [}\begin{array}{c} Avg-Fitness=\frac{1}{N}\sum_{i=1}^{N}fitness(i)\# \end{array}\mathrm{\backslash ]}$$ (14)

where *N* is the number of trial tours and fitness(i) is the fitness value of the best solution generated in the *ith* time.

2. Average classification accuracy: With the help of this evaluation metric, we can determine the proportion of the examined cases in the test dataset that are accurately classified from N runs, which is calculated as follows:

$$\begin{array}{c} \begin{array}{c} Avg-Accuracy=\frac{1}{N}\sum_{i=1}^{N}Accuracy(i) \end{array} \end{array}$$ (15)

$$\begin{array}{c} \begin{array}{c} Accuracy=\frac{TP+TN}{TP+TN+FP+FN} \end{array} \end{array}$$ (16)

where Accuracy(i) denotes the categorization accuracy of the highest quality solution generated at run *i*. The category is defined as the category that was rejected. where *TN* denotes a rejected category, *FP* denotes a misrecognized category, *FN* is a false negative indicating a wrongly rejected category, and *TP* is a true positive indicating dissatisfaction with a correctly defined category.

3. Average sensitivity: The purpose of this assessment method is to determine the percentage of all true positive instances in the test dataset over N experimental runs using equation 17.

$$\begin{array}{c} \begin{array}{c} Avg-Sensitivity=\frac{1}{N}\sum_{i=1}^{N}Sensitivity(i) \end{array} \end{array}$$ (17)

$$\begin{array}{c} \begin{array}{c} Sensitivity=\frac{TP}{TP+FN} \end{array} \end{array}$$ (18)

where the sensitivity result of the best solution generated by the *ith* run is denoted by Sensitivity(i).

4. Average specificity: This metric is used to determine the proportion of all true-negative cases in the test dataset in run N, as shown below:

$$\begin{array}{c} \begin{array}{c} Avg-Specificity=\frac{1}{N}\sum_{N}^{1}Specificity(i) \end{array} \end{array}$$ (19)

$$\begin{array}{c} \begin{array}{c} Specificity=\frac{TN}{FP+TN} \end{array} \end{array}$$ (20)

where Specificity(i) denotes the specificity result of the optimal solution produced by the *ith* run.

5. Average number of chosen features: This metric aims to give the average size of feature selection over N runs. It is calculated by the formula:

$$\begin{array}{c} \begin{array}{c} Avg-FeatNumber=\frac{1}{N}\sum_{i=1}^{N}\frac{F(i)}{M} \end{array} \end{array}$$ (21)

where *M* is the total number of features in the dataset under consideration and F(i) is the number of features selected among the best solutions with minimum fitness values generated by run *i*.

6. Average F-score: is a metric used to evaluate the performance of a classification model, which integrates the two aspects of Precision and Recall. The formula is as follows:

$$\begin{array}{c} \begin{array}{c} \left\{ \begin{array}{c} Pre=\frac{TP}{TP+FP}\\ Rec=\frac{TP}{TP+FN}\\ F-score=2\times \frac{Pre\times Rec}{Pre+Rec} \end{array}\right.\; \end{array} \end{array}$$ (22)

$$\mathrm{\backslash [}\begin{array}{c} Avg-F=\frac{1}{N}\sum_{i=1}^{N}F-score(i)\# \end{array}\mathrm{\backslash ]}$$ (23)

#### 5.2.2. Statistical test.

To verify the superior performance of our proposed algorithm, IRBMO (Improved Random-Based Multi-Objective Optimizer), in all aspects, in this section, we have carefully adopted the Wilcoxon rank sum test, a statistical method. aimed at assessing in depth whether the performance of IRBMO in each run is significantly different from the other algorithms involved in the comparison at the P = 5% significance level. These algorithms include: Differential Evolutionary Algorithm (DE) [30], Genetic Algorithm (GA) [61], Goose Optimization Algorithm (GOOSE) [62], Gray Wolf Optimizer (GWO) [32], Parrot Optimizer (PO) [63], Whale Optimization Algorithm (WOA) [31], Artificial Rabbits Optimization (ARO) [36], Capuchin Search Algorithm(CAPSA) [35], Electric Eel Foraging Optimization Algorithm (EEFO) [29], Elk herd Optimizer(EHO) [64], White Shark Optimizer (WSO) [34] and Covariance Matrix Adaptation Evolution Strategy (CMAES) [65]. To ensure the fairness and credibility of the experimental results, we finely tuned and optimized the key parameters of all the algorithms involved in the comparison, and the specific parameter settings are shown in Table 6 and Table 13.

This testing process strictly follows the standard practice in the academic literature, in which the original hypothesis H0 is set as there is no significant difference between the two algorithms. Specifically, if the P-value of the test is less than 5%, we have good reasons to reject the original hypothesis, which signifies that there is indeed a significant performance difference between IRBMO and the comparison algorithm; on the contrary, if the P-value is greater than 5%, we accept the original hypothesis, i.e., we believe that the two algorithms perform similarly, and it is difficult to distinguish between their strengths and weaknesses, and the “NaN” value reflects this situation. The “NaN” value reflects this situation, which means that the performance of the two algorithms is too close to be compared effectively.

In Table 4, we detail the test results of IRBMO with dimension 30 in the CEC-2017 test set, and make a comprehensive comparison with other well-known comparison algorithms in the field. In order to present the comparison results more intuitively, we have specifically bolded the values with P-values greater than 0.05 (i.e., not reaching the significance level). It is worth noting that there is no “NaN” value in the results of CEC-2017, a phenomenon that indicates that the optimization results of SBOA (another optimization algorithm) are usually able to produce significantly different performances from other algorithms, and thus have a certain degree of differentiation. Looking further at the data in the table, we can see that algorithms like ARO and DE do not show particularly outstanding data performance in the CEC-2017 results, and their data points are rarely highlighted in bold form display. In contrast, IRBMO exhibits significant differences from DE and other metaheuristic algorithms, which further confirms IRBMO’s superiority in performance.

**Table 4:** P-value on CEC-2017(Dim = 30).

Function	ARO	CapSA	CMAES	DE	EEFO	EHO	GA	GOOSE	GWO	PO	WOA	WSO
F1	3.02E-11	3.02E-11	3.02E-11	1.21E-12	1.21E-12	1.47E-07	3.02E-11	1.47E-07	3.02E-11	6.70E-11	3.02E-11	**5.40E-01**
F2	3.02E-11	3.02E-11	3.02E-11	1.21E-12	1.21E-12	2.16E-03	1.61E-10	9.88E-03	3.02E-11	5.49E-11	3.02E-11	**4.55E-01**
F3	3.02E-11	3.02E-11	3.02E-11	1.21E-12	1.21E-12	2.77E-05	3.02E-11	5.26E-04	3.02E-11	1.21E-10	6.70E-11	**3.87E-01**
F4	3.02E-11	3.02E-11	3.02E-11	3.35E-08	1.21E-12	5.22E-12	3.02E-11	**8.77E-02**	3.02E-11	4.08E-11	3.65E-08	7.24E-02
F5	3.02E-11	3.02E-11	3.02E-11	4.08E-05	1.21E-12	4.36E-02	3.02E-11	1.73E-06	3.02E-11	8.99E-11	3.02E-11	6.67E-03
F6	3.02E-11	3.02E-11	3.02E-11	3.36E-11	1.21E-12	3.02E-11	3.02E-11	1.69E-09	3.02E-11	4.98E-11	3.02E-11	**7.73E-01**
F7	3.02E-11	3.02E-11	3.02E-11	9.53E-07	1.21E-12	3.67E-03	3.02E-11	**7.01E-02**	3.02E-11	3.02E-11	5.49E-11	**1.12E-01**
F8	3.02E-11	3.02E-11	3.02E-11	3.02E-11	1.21E-12	3.02E-11	4.98E-11	3.02E-11	5.97E-09	3.02E-11	3.02E-11	**5.49E-01**
F9	1.21E-12	1.21E-12	1.21E-12	1.21E-12	1.21E-12	3.64E-02	3.47E-10	2.84E-04	1.82E-09	4.50E-11	1.21E-12	**7.98E-02**
F10	1.21E-12	3.02E-11	1.21E-12	1.21E-12	1.21E-12	2.11E-11	3.02E-11	**5.01E-01**	2.76E-11	3.69E-11	1.81E-11	**1.02E-01**
F11	1.21E-12	1.72E-12	1.21E-12	1.21E-12	1.21E-12	7.77E-09	3.02E-11	**4.73E-01**	1.65E-11	3.47E-10	1.93E-12	**2.71E-01**
F12	3.02E-11	3.02E-11	3.02E-11	9.30E-04	1.21E-12	**1.41E-01**	5.49E-11	4.64E-05	3.02E-11	2.15E-10	3.02E-11	**5.40E-01**
F13	3.02E-11	3.02E-11	3.02E-11	3.02E-11	1.21E-12	2.43E-05	3.02E-11	5.09E-08	3.02E-11	3.02E-11	3.02E-11	7.70E-04
F14	3.15E-12	1.80E-11	7.04E-07	2.95E-11	1.21E-12	**4.60E-01**	1.64E-05	9.88E-11	**1.96E-01**	**1.09E-01**	**6.20E-01**	2.43E-11
F15	1.21E-10	1.17E-09	7.39E-11	3.34E-03	1.21E-12	**9.94E-01**	2.60E-08	**4.83E-01**	5.46E-06	**3.33E-01**	5.53E-08	2.99E-11
F16	1.46E-11	5.14E-12	**7.73E-02**	3.02E-11	1.21E-12	1.72E-12	1.96E-10	3.99E-04	3.02E-11	1.99E-02	3.02E-11	2.12E-11
F17	1.21E-12	1.21E-12	1.44E-03	1.21E-12	1.21E-12	1.21E-12	3.02E-11	3.02E-11	3.02E-11	2.92E-02	3.02E-11	9.40E-12
F18	3.16E-12	2.97E-11	6.72E-10	1.21E-12	1.21E-12	2.90E-11	**4.29E-01**	9.51E-06	1.31E-08	4.44E-07	1.69E-09	2.33E-11
F19	1.72E-12	6.43E-12	**8.24E-02**	4.50E-11	1.21E-12	1.21E-12	3.02E-11	3.02E-11	2.23E-09	2.13E-05	1.06E-03	2.36E-12
F20	3.00E-10	3.82E-10	4.20E-10	1.61E-10	1.21E-12	7.28E-06	3.34E-11	8.10E-10	1.36E-07	3.08E-08	5.56E-04	4.45E-11
F21	3.24E-11	4.96E-11	3.02E-11	2.21E-06	1.21E-12	1.21E-04	7.38E-10	**4.92E-01**	4.31E-08	9.51E-06	1.19E-06	7.10E-08
F22	2.37E-12	2.98E-11	3.02E-11	1.54E-04	1.21E-12	4.14E-02	7.39E-11	**3.40E-01**	6.07E-11	1.25E-05	2.43E-05	3.53E-09
F23	1.65E-09	3.01E-11	3.02E-11	9.30E-04	1.21E-12	**8.41E-01**	4.50E-11	**6.35E-02**	7.12E-09	9.79E-05	1.17E-04	9.31E-12

To evaluate the performance of the IRBMO algorithm more comprehensively, we also used the nonparametric Friedman mean rank test to rank the experimental results of IRBMO and other algorithms on the CEC-2017 test set. The results show (Table 5) that IRBMO consistently tops the list in terms of average rank, a result that is certainly a strong proof that our proposed optimizer outperforms other benchmark algorithms on the test set under consideration. In summary, IRBMO not only excels in individual performance metrics, but also demonstrates unrivaled superiority in comprehensive performance evaluation.

**Table 5:** Friedman average rank sum test results.

Function	ARO	CapSA	CMAES	DE	EEFO	EHO	GA	GOOSE	GWO	PO	WOA	WSO	IRBMO
F1	7	3	2	5	6	8	13	11	4	9	10	12	**1**
F2	7	3	2	5	6	9	13	11	4	8	12	10	**1**
F3	5	3	2	**1**	6	11	13	9	12	7	10	8	4
F4	4	2	**1**	7	5	13	12	8	11	6	10	9	3
F5	3	2	**1**	9	5	10	13	11	6	7	8	12	4
F6	3	2	**1**	10	8	4	13	12	7	9	6	11	5
F7	3	2	**1**	8	6	9	13	12	7	5	10	11	4
F8	3	2	13	12	8	**1**	11	10	5	7	6	9	4
F9	2	3	5	4	7	11	13	10	6	8	12	9	**1**
F10	7	3	**1**	4	6	13	12	9	5	8	10	11	2
F11	5	2	4	3	7	9	13	11	6	8	12	10	**1**
F12	3	2	**1**	8	6	9	13	11	5	7	12	10	4
F13	3	2	**1**	7	9	10	13	11	6	8	5	12	4
F14	3	6	**1**	13	11	5	7	8	9	10	12	4	2
F15	3	5	**1**	12	6	11	13	10	7	8	9	2	4
F16	4	8	13	11	6	**1**	12	9	5	10	7	3	2
F17	3	9	**1**	12	7	4	13	10	8	11	6	5	2
F18	5	12	**1**	13	8	4	10	9	7	11	6	3	2
F19	4	8	13	11	6	**1**	12	9	7	10	5	3	2
F20	3	5	13	11	6	4	12	9	8	10	7	**1**	2
F21	3	4	13	9	5	6	12	11	7	8	10	2	**1**
F22	3	5	13	10	4	7	12	11	9	8	6	2	**1**
F23	3	4	13	9	5	7	12	11	6	8	10	**1**	2
Avg-Rank	3.87	4.22	5.09	8.43	6.48	7.26	12.17	10.13	6.83	8.30	8.74	6.96	2.52
Overall-rank	2	3	4	10	5	8	13	12	6	9	11	7	**1**

### 5.3. Experiments comparing IRBMO and metaheuristic algorithms

To comprehensively analyze the efficacy and stability of the improved IRBMO algorithm in feature selection tasks, we have crafted a comparative study with nine classical and cutting-edge meta-heuristic algorithms in a systematic manner. These algorithms include Differential Evolutionary Algorithm (DE) [30], Genetic Algorithm (GA) [61], Goose Optimization Algorithm (GOOSE) [62], Gray Wolf Optimizer (GWO) [32], Parrot Optimizer (PO) [63], Whale Optimization Algorithm (WOA) [31], Elk herd Optimizer(EHO) [64], White Shark Optimizer (WSO) [34], Covariance Matrix Adaptation Evolution Strategy (CMAES) [65]. These algorithms are chosen for comparison mainly based on their wide application within the optimization field, their excellent performance and the extent to which they are frequently cited in the literature. In addition, each of these algorithms has its own characteristics and can comprehensively cover the main types and strategies of current optimization algorithms, thus providing a comprehensive and fair evaluation environment for our IRBMO algorithms.

**Fig 4:**
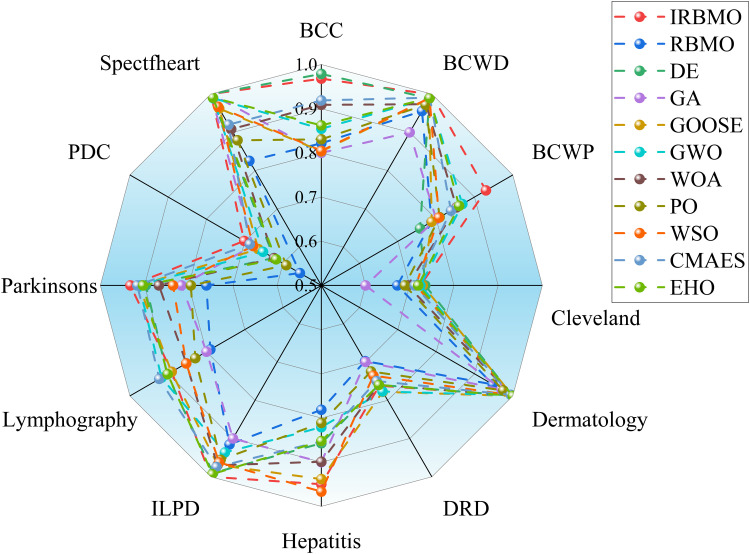
Radar chart of classification accuracy of IRBMO vs. other algorithms.

**Fig 5:**
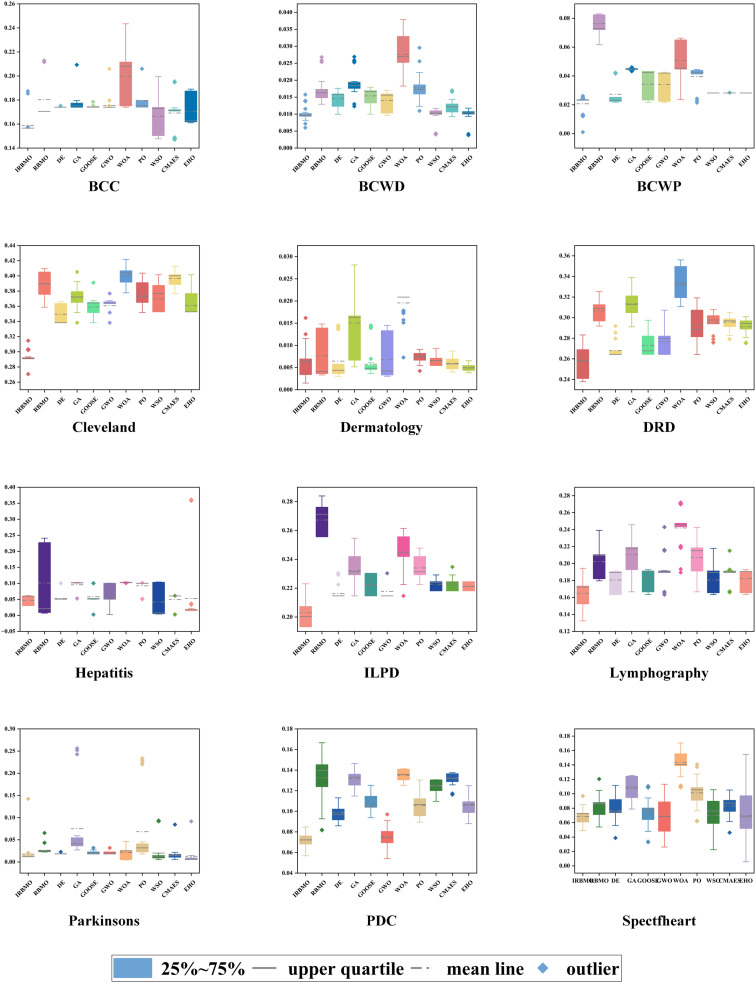
IRBMO vs. meta-heuristic algorithms boxplot.

To maintain experimental fairness and the credibility of the results, we carefully tuned the key parameters of all the compared algorithms, and the detailed parameter configurations are presented in Table 6. During the performance evaluation, we strictly unified the experimental conditions, initialized all the algorithms with 30 population individuals, and ran 100 iterations to complete the optimization process. To enhance the statistical reliability of the results, each algorithm performs 30 independent runs to capture the performance fluctuations and calculate the mean and standard deviation of the algorithms, as shown in Table 7 is the mean and standard deviation of the fitness values recorded. The evaluation metrics cover fitness value, classification accuracy, sensitivity, specificity, number of selected features, and F-score, which not only reveal the average performance of the algorithms, but also quantify their stability through standard deviation. To present the experimental results more intuitively, we use box-and-line plots to visualize and analyze the data distribution characteristics. This presentation makes the performance fluctuations and extremes of each algorithm on different datasets visible at a glance, which helps to provide an in-depth interpretation of the stability and robustness of the algorithms. As shown in Fig 4, the IRBMO algorithm performs particularly well on various metrics, and its fluctuation range and outlier distribution reflect excellent robustness. In addition, we have drawn radar plots of classification prediction accuracy to present the effectiveness of various algorithms in classification prediction tasks in a more intuitive way. As shown in Fig 5, the experimental results indicate that the IRBMO algorithm significantly outperforms the other compared algorithms in the feature selection task.

**Table 6:** Comparison of metaheuristic algorithm parameterization.

Algorithm	Parameter settings
DE	CR=0.1; F=0.4
GA	pc=0.8; pm=0.05
GOOSE	coe: It varies randomly in the range [0,0.17]; S_S =343.2
GWO	a: Linear reduction from 2 to 0
WOA	a: Linear reduction from 2 to 0; a2: Linearly increasing from -1–0
PO	a: It varies randomly in the range [0,0.1]
EHO	MalesRate=0.2; rd: It varies randomly in the range [-2,^2^]
WSO	fmin =0.01; fmax=0.98; a0 =10; a1 =100; a2 =0.001
CMAES	μ=n/2; w =log(μ+0.5)−log(1:μ); σ=60

**Table 7:** Fitness values of IRBMO vs. other binary algorithms.

Dataset		IRBMO	RBMO	DE	GA	GOOSE	GWO	WOA	PO	WSO	CMAES	EHO
BCC	Mean	**0.158769**	0.18031	0.17406	0.177484	0.174393	0.175383	0.199771	0.179649	0.166428	0.169152	0.170749
	Std	0.00751	0.018035	0.000203	0.008787	0.000891	0.005865	0.022241	0.010666	0.014408	0.011321	0.012326
BCWD	Mean	**0.01**	0.017218	0.014562	0.019418	0.015385	0.014075	0.027589	0.017767	0.010085	0.012181	0.010006
	Std	0.001838	0.003789	0.002493	0.003633	0.002436	0.002733	0.005497	0.003557	0.001718	0.001845	0.001728
BCWP	Mean	**0.020702**	0.07628	0.027293	0.044637	0.03433	0.03416	0.050525	0.039541	0.0282	0.02821	0.0282
	Std	0.005947	0.00631	0.008219	0.000585	0.00999	0.009844	0.010689	0.007561	1.41E-17	4.06E-5	1.41E-17
Cleveland	Mean	**0.291976**	0.38949	0.349516	0.372377	0.359086	0.360716	0.39999	0.375242	0.36976	0.39682	0.36109
	Std	0.009189	0.01319	0.012564	0.014667	0.014191	0.012443	0.013517	0.013596	0.01737	0.01047	0.01495
Dermatology	Mean	0.006288	0.00766	0.006458	0.015055	0.005734	0.006769	0.019557	0.007354	0.0066	0.00593	**0.00496**
	Std	0.003327	0.00511	0.004318	0.007105	0.002952	0.004644	0.00295	0.001114	0.00108	0.00144	6.14E-4
DRD	Mean	**0.25758**	0.30659	0.26805	0.313338	0.273043	0.276742	0.333696	0.293079	0.29488	0.29565	0.29107
	Std	0.015365	0.01022	0.007345	0.012647	0.011322	0.012643	0.014801	0.014738	0.01001	0.00667	0.00867
Hepatitis	Mean	0.04733	0.10101	0.052571	0.095382	0.058135	0.064296	0.102649	0.092171	**0.04184**	0.04914	0.0527
	Std	0.015016	0.10775	0.008988	0.016951	0.024716	0.025501	0.000756	0.018506	0.04731	0.02365	0.10389
ILPD	Mean	**0.202917**	0.26727	0.216187	0.232141	0.221928	0.217786	0.243854	0.234023	0.22273	0.22178	0.22128
	Std	0.009801	0.01087	0.004202	0.011598	0.007063	0.006369	0.013587	0.006752	0.00299	0.00407	0.00322
Lymphography	Mean	**0.165096**	0.20223	0.180561	0.210527	0.183903	0.191171	0.241984	0.20724	0.18061	0.19106	0.18219
	Std	0.015804	0.01803	0.012945	0.018251	0.011696	0.018593	0.020463	0.01843	0.01607	0.01197	0.01233
Parkinsons	Mean	0.017808	0.02662	0.018485	0.074981	0.021212	0.020455	0.021288	0.068371	0.02019	0.01756	**0.01295**
	Std	0.023656	0.00888	0.001153	0.079604	0.003837	0.003101	0.013315	0.080512	0.02489	0.01856	0.02154
PDC	Mean	**0.072074**	0.1325	0.098834	0.13204	0.107185	0.074407	0.134951	0.105754	0.12429	0.1319	0.10588
	Std	0.006348	0.02128	0.007369	0.007439	0.008214	0.009335	0.00519	0.01024	0.00572	0.00554	0.00779
Spectfheart	Mean	**0.068515**	0.08588	0.076582	0.107621	0.074932	0.068956	0.143432	0.101326	0.06995	0.08304	0.06965
	Std	0.011386	0.0136	0.016009	0.015218	0.019064	0.020727	0.016927	0.018549	0.02357	0.01393	0.03343
(W|T|L)		(9|3|0)	(0|12|0)	(0|12|0)	(0|11|1)	(0|12|0)	(0|12|0)	(0|3|9)	(0|12|0)	(1|11|0)	(0|12|0)	(2|10|0)
Mean		1.50	9.33	3.58	9.25	5.25	5.00	10.58	8.08	4.50	5.33	3.50
Rank		**1**	10	3	9	6	5	11	8	4	7	2

To validate the superiority of the IRBMO algorithm in a multi-dimensional manner, we combine numerical and visual analyses to provide a comprehensive and in-depth evaluation of its performance on multiple datasets. This comprehensive analytical approach not only clearly demonstrates the outstanding performance of the IRBMO algorithm in the feature selection task, but also further confirms its significant advantages in terms of efficacy and robustness.

Careful analysis of Table 7 shows that IRBMO performs the best in the fitness value test, has the lowest average fitness value, and achieves the optimal results in 9 datasets without performing the worst in any dataset. Its overall ranking is 1.50, which is the first place among all the compared algorithms, showing excellent optimization ability and stability. Specifically, IRBMO has the lowest average fitness values on the BCC (0.1588), BCWD (0.0100), and BCWP (0.0207) datasets, which are significantly better than the comparison algorithms. Meanwhile, their standard deviations are generally small, such as BCWD (0.0018), Dermatology (0.0033), and PDC (0.0063), which indicates that the algorithms’ results are stable and have low volatility. In contrast, RBMO, GA and WOA performed poorly, ranking 10, 9 and 11, respectively. RBMO did not win on all datasets and achieved the highest fitness values on several datasets, e.g., BCWP (0.0763), Cleveland (0.3895) and DRD (0.3066), suggesting a limited ability to optimize. WOA achieves better results on only 3 datasets but performs poorly on the remaining 9 datasets with high fitness values, e.g., Spectfheart (0.1434). GA achieves high fitness values on a number of datasets such as BCWD, BCWP, and DRD and ranks 9th with weak overall performance. EHO and DE rank 2nd and 3rd with sub-optimal performance. EHO achieves the lowest fitness values on the Dermatology (0.00496) and Parkinsons (0.01295) datasets, with better overall stability, but still has higher fitness values than IRBMO on some datasets, such as DRD (0.2911) and Spectfheart (0.0697).DE achieves higher fitness values on the BCWP, Parkinsons and PDC datasets, but still lags behind IRBMO in average ranking.

To present the performance advantages of the IRBMO algorithm visually, we visualized the fitness values by means of box-and-line plots (see Fig 5). The box-and-line plot analysis shows that IRBMO achieves the lowest or near-minimum fitness values on multiple datasets, demonstrating excellent optimization capabilities. Meanwhile, its small standard deviation and compact box range indicate that the algorithm has strong stability and robustness. On the BCC, BCWD, BCWP, Cleveland, ILPD, and Parkinsons datasets, IRBMO adaptation values are significantly lower than those of other algorithms, and the distribution is more concentrated, which further proves the stability of its optimization effect. In contrast, EHO, DE and CMAES perform better on individual datasets, for example, DE has lower adaptation values on the BCWP dataset, but the overall fluctuation is larger and the stability is insufficient. GWO and GOOSE achieve better optimization results on some datasets, but the distribution of the adaptation values is wider and the performance is not robust enough. The algorithms WSO, WOA, GA, RBMO and PO have high overall adaptation values, especially RBMO performs the worst on multiple datasets with more outliers, indicating that its optimization ability is limited and it is difficult to adapt to the characteristics of different datasets.

Careful analysis of Table 8 reveals that the IRBMO algorithm exhibits a notable performance advantage in terms of classification accuracy metrics. It boasts a mean rank of 2.25 and a comprehensive ran of 1. IRBMO achieves the highest classification accuracy on 6 out of 12 datasets and does not have the worst performance on any dataset, while its standard deviation was generally low, further demonstrating its stability and robustness. EHO ranked second with an average ranking of 4.17, performing optimally on 1 dataset but intermediate on most datasets. DE and CMAES were ranked third and fourth with average rankings of 4.25 and 4.75, respectively, with DE performing optimally on 3 datasets but worst on the BCWP dataset, and CMAES performing optimally on 1 dataset but centrally on multiple datasets. GWO and GOOSE were ranked fifth and sixth with mean rankings of 4.75 and 4.83, respectively, with GWO performing optimally on 1 dataset and GOOSE performing optimally on 2 datasets, but both were intermediate on most datasets. WSO, WOA, and GA performed poorly, with average rankings of 6.00, 6.42, and 8.25, respectively, with WSO performing optimally on the Hepatitis dataset, and WOA and GA performing optimally on 1 dataset each, but poorly on multiple datasets. RBMO and PO performed the worst, with mean rankings of 10.25 and 8.92, respectively, with RBMO performing the worst on 6 datasets and PO performing centrally on all datasets but with generally low accuracy.

**Table 8:** Classification accuracy values of IRBMO vs. other binary algorithms.

Dataset		IRBMO	RBMO	DE	GA	GOOSE	GWO	WOA	PO	WSO	CMAES	EHO
BCC	Mean	0.968	0.821667	**0.978667**	0.8	0.803333	0.854667	0.908667	0.830667	0.804667	0.918667	0.862
	Std	0.010635	0.016206	0.01432	3.39E-16	0.015162	0.017564	0.022397	0.016386	0.007303	0.008604	0.010635
BCWD	Mean	**0.999**	0.955667	0.989	0.9	0.987667	0.989	0.973667	0.972	0.99	0.990667	0.99
	Std	0.003051	0.00504	0.003051	4.52E-16	0.006261	0.004807	0.008503	0.004842	5.65E-16	0.003651	5.65E-16
BCWP	Mean	**0.93**	0.787667	0.757667	0.8	0.788333	0.868333	0.853	0.809	0.807	0.838667	0.86
	Std	2.26E-16	0.021121	0.017357	3.39E-16	0.009499	0.015332	0.015347	0.025373	0.041866	0.017167	3.39E-16
Cleveland	Mean	0.724333	0.672	**0.735**	0.6	0.729333	0.724333	0.695333	0.689	0.713667	0.711	0.719
	Std	0.006261	0.011265	0.013326	0	0.023183	0.005683	0.010417	0.01269	0.009994	0.00712	0.013481
Dermatology	Mean	0.997667	0.948	0.999333	0.970333	**1**	0.996667	0.998	0.975333	0.997	0.995	**1**
	Std	0.004302	0.010306	0.002537	0.029767	0	0.004795	0.004068	0.01008	0.004661	0.005085	0
DRD	Mean	0.762	0.697667	0.76	0.7	**0.778**	0.777	0.76	0.725	0.735667	0.75	0.76
	Std	0.023253	0.018511	3.39E-16	3.39E-16	0.004842	0.011788	0.009469	0.019957	0.0104	0	3.39E-16
Hepatitis	Mean	0.95	0.781333	0.857333	0.9	0.938333	0.821	0.899	0.811333	**0.966667**	0.853	0.852
	Std	4.52E-16	0.008996	0.007397	4.52E-16	0.023937	0.020231	0.020401	0.009371	0.008442	0.013684	0.010306
ILPD	Mean	**1**	0.915667	0.990333	0.9	0.969	0.936667	0.97	0.955667	0.962667	0.974333	0.993333
	Std	0	0.020792	0.010334	4.52E-16	0.010939	0.009223	0.010505	0.016543	0.019106	0.008976	0.008841
Lymphography	Mean	0.891	0.79	0.902667	0.8	0.891667	0.917667	0.852667	0.829333	0.853	**0.923333**	0.901667
	Std	0.008847	0.008305	0.007397	3.39E-16	0.011167	0.012507	0.008683	0.009072	0.008367	0.004795	0.017436
Parkinsons	Mean	**0.932**	0.76	0.897	0.816667	0.912667	0.912333	0.867667	0.795333	0.835667	0.916333	0.903667
	Std	0.016897	0.021656	0.012905	0.030663	0.013113	0.01775	0.016543	0.019605	0.025554	0.01129	0.022664
PDC	Mean	**0.701333**	0.556	0.624	0.679333	0.664667	0.652333	0.623667	0.591667	0.678333	0.687	0.618333
	Std	0.024877	0.019582	0.011017	0.040252	0.013322	0.008976	0.014735	0.027175	0.012341	0.012077	0.005307
Spectfheart	Mean	**1**	0.825667	**1**	**1**	0.962333	0.989	0.908	0.879333	0.967	0.92	0.990667
	Std	0	0.024731	0	0	0.022542	0.013983	0.020578	0.024626	0.031639	0.025731	0.011427
(W|T|L)		(6|6|0)	(0|5|7)	(3|8|1)	(1|7|4)	(2|10|0)	(0|12|0)	(0|12|0)	(0|12|0)	(1|11|0)	(1|11|0)	(1|11|0)
Mean		**2.25**	**10.42**	**4.33**	**8.25**	**4.92**	**5.08**	**6.33**	**8.83**	**6.00**	**4.67**	**4.42**
Rank		1	11	2	9	5	6	8	10	7	4	3

To more intuitively demonstrate the performance of each algorithm in terms of classification accuracy, we plotted a radar chart of classification accuracy. It can be clearly observed from Fig 4 that the performance of IRBMO algorithm is particularly outstanding, and its contours are located on the outermost side on several datasets, which intuitively demonstrates its significant advantages on the 12 datasets. IRBMO achieves the highest classification accuracy on 6 datasets, and the overall contours are smooth and less fluctuating, which further corroborates its stability and robustness. In contrast, the contours of the other algorithms perform differently on different datasets: the contours of EHO, DE and CMAES are closer but significantly lower than those of IRBMO on some datasets, especially on the BCWP and BCC datasets, where the performance of DE and CMAES decreases. The profiles of GWO and GOOSE are close to IRBMO in some datasets, but still have some gap in most datasets, especially in DRD and Dermatology datasets, where GOOSE performs better but fluctuates more overall. The profiles of WSO, WOA and GA are significantly inward-looking, especially on the Hepatitis and BCC datasets, and the overall accuracy is low. RBMO and PO have the most inward-looking profiles, especially on the BCC and PDC datasets, and the overall performance is significantly lower than that of the other algorithms. The radar charts visualize that IRBMO leads the classification accuracy in all aspects, and the coverage and stability of its contours are better than the other algorithms, which further validates its efficiency and reliability in the classification task. Other algorithms such as EHO, DE, and CMAES perform moderately well, while WSO, WOA, GA, PO, and RBMO perform poorly, especially on multiple datasets with significantly lower accuracy.

Careful analysis of Table 9 shows that the CMAES algorithm performs optimally in the comparison of sensitivity metrics, with an average ranking of 3.00 and an overall ranking of first place. It achieved the highest sensitivity on 5 datasets such as BCWD (1.000), Dermatology (1.000) and ILPD (0.994333) with low standard deviation and high stability. IRBMO followed with a mean ranking of 3.75 and ranked second, with the best performance on 4 datasets such as BCC (0.744333) and Hepatitis (1.000). GWO,EHO ranked third and fourth with mean rankings of 4.42 and 4.50 respectively. GWO performed optimally on DRD (0.951333) and Lymphography (0.991667), and EHO performed optimally on BCWD (1.000) and Spectfheart (0.954667). DE was ranked fifth with a mean ranking of 4.83, performing optimally on 3 datasets but poorly on BCWP (0.530) and PDC (0.251667). GA, GOOSE, and WOA performed moderately well with mean rankings of 5.08, 5.25, and 6.58, respectively. WSO, PO, and RBMO performed poorly with mean rankings of 6.67, 9.25, and 9.67, respectively, with PO performing the worst on BCC (0.167) and Spectfheart (0.446333), and RBMO performing the worst on 6 datasets. sets where PO performed worst on BCC (0.398333) and Spectfheart (0.607333). The experimental results show that CMAES significantly outperforms other algorithms in sensitivity metrics, IRBMO, GWO and EHO perform well, DE, GA, GOOSE and WOA perform moderately, and WSO, PO and RBMO perform poorly.

**Table 9:** Sensitivity values of IRBMO vs. other binary algorithms.

Dataset		IRBMO	RBMO	DE	GA	GOOSE	GWO	WOA	PO	WSO	CMAES	EHO
BCC	Mean	0.744333	0.398333	**0.842333**	0.325333	0.385	0.508	0.442667	0.167	0.258333	0.575333	0.478
	Std	0.07646	0.067573	0.128135	0.059927	0.046886	0.058628	0.114136	0.072214	0.035339	0.046292	0.046713
BCWD	Mean	0.999	0.99	**1**	0.995	**1**	0.995667	0.990333	0.998333	0.999333	**1**	**1**
	Std	0.003051	5.65E-16	0	0.005085	0	0.006789	0.008899	0.00379	0.002537	0	0
BCWP	Mean	**0.860667**	0.76	0.53	0.794333	0.85	0.768	0.780667	0.712667	0.732667	0.811	0.822333
	Std	0.05711	3.39E-16	2.26E-16	0.036073	3.39E-16	0.136493	0.02947	0.085458	0.088002	0.07208	0.064149
Cleveland	Mean	0.641333	0.585667	0.650333	0.715	0.613667	0.655667	0.701333	0.578	0.588	**0.722667**	0.63
	Std	0.031594	0.046437	0.021732	0.020469	0.044912	0.024731	0.021129	0.031448	0.027342	0.030505	0.027792
Dermatology	Mean	**1**	0.97	**1**	**1**	**1**	**1**	0.993667	0.993	**1**	**1**	**1**
	Std	0	4.52E-16	0	0	0	0	0.007184	0.009523	0	0	0
DRD	Mean	0.875333	0.816333	0.86	0.861333	0.843667	**0.951333**	0.895	0.855	0.899	0.86	0.85
	Std	0.017367	0.019737	3.39E-16	0.01008	0.014016	0.027759	0.040493	0.019253	0.032732	3.39E-16	3.39E-16
Hepatitis	Mean	**1**	0.914333	0.977	0.954	0.950667	0.971	0.964	0.955	0.929	0.967333	0.962667
	Std	0	0.013047	0.012635	0.010034	0.01388	0.013481	0.014288	0.018892	0.018261	0.009803	0.008683
ILPD	Mean	**1**	0.935	0.993	0.959333	0.977333	0.988333	0.982667	0.975	0.968667	0.994333	0.995333
	Std	0	0.013582	0.007944	0.009444	0.013374	0.010854	0.015071	0.016135	0.02255	0.007279	0.006814
Lymphography	Mean	0.985333	0.940333	0.977	0.993333	0.987667	0.991667	0.99	0.945667	0.982667	**0.995333**	0.994
	Std	0.009732	0.003198	0.010554	0.006065	0.004302	0.007466	5.65E-16	0.007739	0.007849	0.005713	0.006215
Parkinsons	Mean	0.965333	0.873	0.965667	0.907	0.926667	0.959	0.907	0.832333	0.89	**0.968667**	0.938333
	Std	0.01925	0.016006	0.022079	0.018597	0.01647	0.022183	0.029496	0.043206	0.037509	0.025289	0.033019
PDC	Mean	0.275667	0.358667	0.251667	**0.534**	0.401667	0.365	0.288	0.304333	0.512333	0.463	0.255333
	Std	0.158216	0.090696	0.039136	0.214261	0.115104	0.089587	0.073127	0.153526	0.141218	0.122563	0.118168
Spectfheart	Mean	0.899667	0.607333	0.819333	**1**	**1**	0.685	0.465667	0.446333	0.775667	0.647333	0.954667
	Std	0.081091	0.218931	0.184614	0	0	0.313751	0.135715	0.10384	0.244677	0.091874	0.075691
(W|T|L)		(4|8|0)	(0|6|6)	(3|7|2)	(3|9|0)	(3|9|0)	(2|10|0)	(0|12|0)	(0|8|4)	(1|11|0)	(5|7|0)	(2|10|0)
Mean		3.75	9.67	4.83	5.08	5.25	4.42	6.58	9.25	6.67	3.00	4.50
Rank		2	11	5	6	7	3	8	10	9	**1**	4

Carefully analyzing Table 10, the IRBMO algorithm performs optimally in the comparison of specificity metrics, with a mean rank of 2.08 and the first overall rank. It achieved the highest specificity on five datasets, such as BCC (0.996), ILPD (1.000), and Spectfheart (1.000), with low standard deviation and high stability. GOOSE was ranked second with a Mean ranking of 3.75, and performed optimally on four datasets, such as BCWP (1.000) and Spectfheart (1.000). DE was ranked third with a mean ranking of 3.92, performing optimally on 1 dataset (BCWP, 0.984333) but with intermediate performance on most of the datasets. EHO was ranked fourth with a mean ranking of 4.17, performing optimally on 1 dataset (Spectfheart, 1.000), and with a more balanced performance overall. CMAES was ranked fifth with a mean ranking of 5.75, performing optimally on 3 datasets, such as BCWD (0.995) and PDC (0.906), but poorly on ILPD (0.885333).GWO, GA, and WOA performed moderately well, with mean rankings of 6.83, 7.08, and 6.00, respectively, with GWO performing moderately on DRD (0.252667) and Lymphography (0.660667), GA performed better on BCWP (0.907) and Spectfheart (1.000), and WOA performed moderately well on all datasets. WSO, PO, and RBMO performed poorly with mean rankings of 6.42, 8.50, and 9.75, respectively, with WSO performing optimally on 1 dataset (ILPD, 0.949667), PO performing worst on 3 datasets (e.g., DRD, 0.343), and RBMO performing worst on 5 datasets (e.g., BCC, 0.981 and Cleveland, 0.612). The experimental results show that IRBMO significantly outperforms the other algorithms on specificity metrics, GOOSE, DE, and EHO perform well, CMAES, GWO, GA, and WOA perform moderately well, and WSO, PO, and RBMO perform poorly.

**Table 10:** Specificity values of IRBMO vs. other binary algorithms.

Dataset		IRBMO	RBMO	DE	GA	GOOSE	GWO	WOA	PO	WSO	CMAES	EHO
BCC	Mean	0.996	0.981	0.995333	0.988	**1**	0.98	0.995667	0.993667	0.995	0.988667	0.995667
	Std	0.005632	0.014937	0.006288	0.011861	0	0.015974	0.005683	0.008899	0.007311	0.01383	0.006261
BCWD	Mean	0.99	0.929667	0.959667	0.945	0.954	0.965	0.993	0.926667	0.98	**0.995**	0.967667
	Std	0.009826	0.010334	0.003198	0.014797	0.012205	0.016764	0.008367	0.008841	3.39E-16	0.005085	0.013817
BCWP	Mean	0.945667	0.888	0.984333	0.907	**1**	0.902	0.924333	0.882333	0.898667	0.881	0.906667
	Std	0.049038	0.038632	0.017943	0.027935	0	0.027842	0.042156	0.041496	0.05746	0.040288	0.073594
Cleveland	Mean	**0.852667**	0.612	0.818333	0.63	0.807333	0.800333	0.694	0.816333	0.833667	0.706667	0.814667
	Std	0.016174	0.034381	0.014875	0.033322	0.036382	0.019205	0.037564	0.036811	0.034188	0.02975	0.012794
Dermatology	Mean	**0.986**	0.887	0.958	0.912333	0.956333	0.948667	0.97	0.929	0.954	0.977333	0.978333
	Std	0.006747	0.009154	0.009248	0.007279	0.004901	0.012794	4.52E-16	0.005477	0.006747	0.008277	0.004611
DRD	Mean	**0.586333**	0.440667	0.5	0.459	0.573333	0.252667	0.430667	0.343	0.39	0.54	0.53
	Std	0.084506	0.021645	0	0.022796	0.03273	0.09595	0.104417	0.067013	0.061082	4.52E-16	2.26E-16
Hepatitis	Mean	0.954	0.854333	0.948	0.887	0.901	**0.955667**	0.885333	0.835	0.865333	0.931	0.908667
	Std	0.016526	0.016955	0.019896	0.016006	0.01936	0.022079	0.025015	0.033296	0.044469	0.020231	0.031703
ILPD	Mean	**1**	0.886333	0.978667	0.904	0.939	0.729333	0.894	0.847333	0.949667	0.885333	0.978333
	Std	0	0.062779	0.028129	0.052693	0.046635	0.068929	0.03359	0.082752	0.051225	0.047397	0.027554
Lymphography	Mean	0.670667	0.498	0.644333	0.536	0.599	0.660667	0.525667	0.540333	0.521667	**0.683333**	0.641667
	Std	0.050373	0.022499	0.031588	0.032758	0.036137	0.025989	0.043286	0.02498	0.032279	0.030551	0.05902
Parkinsons	Mean	0.805667	0.65	0.792667	0.703333	**0.831333**	0.635333	0.706	0.596333	0.688	0.6	0.806333
	Std	0.145405	0.058898	0.099062	0.065091	0.115003	0.138059	0.086047	0.15533	0.092266	0.14147	0.081769
PDC	Mean	0.864333	0.792667	0.862333	0.848333	0.899	0.861667	0.871	0.882667	0.804333	**0.906**	0.828667
	Std	0.087363	0.049196	0.041413	0.058314	0.016682	0.024081	0.058801	0.034535	0.043919	0.06355	0.071232
Spectfheart	Mean	**1**	0.932333	**1**	**1**	**1**	**1**	0.990333	0.988333	**1**	0.984667	**1**
	Std	0	0.019945	0	0	0	0	0.012172	0.013412	0	0.014077	0
(W|T|L)		(5|7|0)	(0|7|5)	(1|11|0)	(1|11|0)	(4|8|0)	(2|7|3)	(0|12|0)	(0|9|3)	(1|11|0)	(3|8|1)	(1|11|0)
Mean		2.08	9.75	3.92	7.08	3.75	6.83	6.00	8.50	6.42	5.75	4.17
Rank		**1**	11	3	9	2	8	6	10	7	5	4

Careful analysis of Table 11 shows that the GA algorithm performs optimally in the comparison of the number of features (Number of Selected Features), with an average rank (Mean) of 2.50 and an overall rank (Rank) of first place. It achieved the least number of features (W) on 5 datasets such as Dermatology (1.5), Parkinsons (1.833333) and Spectfheart (2.366667) with low standard deviation and high stability. IRBMO ranked the second with a Mean Rank of 2.67 and performed optimally on 2 datasets such as BCWD (5.233333) and Spectfheart (2.766667). DE ranked third with a mean ranking of 3.83 and performed optimally on 3 datasets such as DRD (2.0), Hepatitis (2.233333), and Spectfheart (1.533333.) GWO ranked fourth with a mean ranking of 4.42 and performed moderately well across all datasets and did not perform optimally or poorly on any dataset. CMAES and EHO tied for fifth place with a mean ranking of 5.17, with CMAES performing optimally on 2 datasets (e.g., ILPD, 3.733333) and EHO performing intermediate on all datasets. GOOSE, WOA, and WSO performed moderately well, with mean rankings of 6.92, 6.75, and 7.92, respectively, with GOOSE performing 1 dataset at the worst (Lymphography, 353.7), WOA on 1 dataset (Lymphography, 273.0333), and WSO on all datasets. PO and RBMO performed poorly, with mean rankings of 9.83 and 10.25, respectively, with PO performing worst on 5 datasets (e.g., BCC 13.33333 and Lymphography, 371.1), and RBMO performed the worst on 5 datasets (e.g., BCC, 13.03333 and Lymphography, 367.2333). The experimental results show that GA significantly outperforms the other algorithms in terms of the number of features, IRBMO, DE, and GWO perform well, CMAES, EHO, GOOSE, WOA, and WSO perform moderately, and PO and RBMO perform poorly.

**Table 11:** Number of features selected for IRBMO vs. other binary algorithms.

Dataset		IRBMO	RBMO	DE	GA	GOOSE	GWO	WOA	PO	WSO	CMAES	EHO
BCC	Mean	5.233333	13.03333	7.133333	**3.366667**	7.833333	5.966667	6.533333	13.33333	11.76667	6.033333	11.16667
	Std	1.774986	2.882568	2.956388	0.490133	1.487496	2.042367	3.298206	2.820288	2.160513	2.456284	3.195723
BCWD	Mean	5.233333	17.03333	7.933333	**3**	10.1	7.7	9.066667	17.56667	11.63333	14.53333	6.366667
	Std	1.330889	5.102287	2.377094	1.875431	1.561388	1.878187	6.356823	6.032574	1.519604	3.339764	1.473521
BCWP	Mean	**2.466667**	4.133333	4	3.433333	4	4.066667	4.333333	4.133333	3.833333	2.6	4
	Std	1.136642	1.655364	0	0.568321	0	1.048261	1.372974	1.382984	0.985527	1.162637	0
Cleveland	Mean	5.5	8.3	**4.5**	5.933333	5.366667	5.633333	7.033333	8.8	8.566667	6.1	7.3
	Std	2.208916	1.393334	1.074789	4.042305	1.542129	2.31164	2.592873	1.769473	2.07918	2.771157	1.087547
Dermatology	Mean	8.033333	19.5	11.53333	**1.5**	14.3	11.23333	17.53333	19.43333	16.73333	16.93333	11.6
	Std	1.217214	3.431598	1.795268	0.508548	2.818045	1.454679	3.491204	2.387949	2.434427	4.940566	2.094327
DRD	Mean	3.3	4.366667	2	3.133333	4	2.966667	3.2	3.466667	3.833333	**1.4**	2
	Std	0.702213	1.691425	0	0.776079	0	0.999425	1.297212	1.279368	1.261727	0.674665	0
Hepatitis	Mean	5.666667	10.03333	**2.233333**	6.833333	8	6.366667	6.9	9.1	9.366667	9.4	6
	Std	0.994236	2.008316	0.568321	1.931291	1.114172	0.850287	3.835856	2.339319	0.999425	3.747183	1.203443
ILPD	Mean	5.4	12.16667	6.366667	5.2	6.366667	4.2	7.6	9.9	7.966667	**3.733333**	5.9
	Std	1.191927	4.136326	1.401559	2.605035	0.668675	1.242911	2.711406	3.72642	1.884297	0.784915	1.422722
Lymphography	Mean	121	367.2333	222.6	**36.63333**	353.7	137.3333	273.0333	371.1	366.4333	145.5333	341.7667
	Std	66.47374	10.52315	37.89878	1.79046	9.858132	21.91729	174.3735	15.35859	12.4836	75.64606	17.72721
Parkinsons	Mean	4.433333	20.66667	11.63333	**1.833333**	19.56667	12.93333	15.7	20.7	19	4.633333	18.73333
	Std	1.250747	2.682425	2.442206	0.461133	2.896887	3.321127	6.131603	3.207212	4.193591	2.092406	4.050827
PDC	Mean	**2.9**	6.266667	6	3.833333	6.566667	5.433333	6.2	6.2	5.9	6.033333	5.066667
	Std	1.061879	1.048261	0.787839	1.839853	1.104328	0.85836	0.886683	0.761124	1.516575	2.326509	1.638614
Spectfheart	Mean	2.766667	8.166667	**1.533333**	2.366667	2.866667	6.633333	2.533333	7.933333	5.866667	3.933333	3.466667
	Std	0.504007	1.984132	0.507416	0.614948	1.136642	1.771096	0.571346	2.016028	1.547709	1.595972	1.195778
(W|T|L)		(2|10|0)	(0|7|5)	(3|9|0)	(5|7|0)	(0|11|1)	(0|12|0)	(0|11|1)	(0|7|5)	(0|12|0)	(2|10|0)	(0|12|0)
Mean		2.67	10.25	3.83	2.50	6.92	4.42	6.75	9.83	7.92	5.17	5.17
Rank		2	11	3	**1**	8	4	7	10	9	5	5

Carefully analyzing Table 12, the IRBMO algorithm demonstrates a significant performance advantage in the comparison of F-score metrics, with a mean rank of 2.92 and an overall rank of No. 1. IRBMO achieves the highest F-scores on four out of the twelve datasets and does not have the worst performance on any dataset, especially on the BCC, Dermatology, ILPD and Spectfheart datasets, while its low standard deviation further demonstrates its stability and robustness. GOOSE ranked second with a mean ranking of 3.92, performing optimally on three datasets (e.g., BCWP, ILPD and Parkinsons datasets) and overall performance. GWO was ranked third with an average ranking of 4.50 and performed optimally on 2 datasets (e.g., DRD and Lymphography datasets) but performed poorly on the PDC dataset. CMAES was ranked fourth with an average ranking of 4.92 and performed optimally on the Parkinsons dataset but performed poorly on the PDC and Spectfheart datasets. DE was ranked fourth with an average ranking of 4.92 and performed optimally on the PDC and Spectfheart datasets. CMAES ranked fourth with an average ranking of 4.92, performing optimally on the Parkinsons dataset but poorly on the PDC and Spectfheart datasets, while DE and EHO tied for fifth with an average ranking of 5.00. DE was the best performer on the BCC, Dermatology, and ILPD datasets but poorly on the Hepatitis and PDC datasets, and EHO was the best performer on the BCWD, ILPD, and Parkinsons datasets, resulting in a more balanced overall performance. GA, WOA and WSO tied for the seventh place with an average ranking of 6.50 respectively, with GA performing best on BCWP and Spectfheart datasets, WOA performing in the middle of all datasets, and WSO performing best on ILPD dataset. RBMO and PO performed poorly with an average ranking of 8.58 and 9.67, with RBMO performing worst on 2 datasets (e.g., BCWD, ILPD and Parkinsons dataset). datasets (e.g., BCC and PDC datasets) and PO performed worst on 5 datasets (e.g., BCC, BCWP, Hepatitis, PDC, and Spectfheart datasets). The experimental results show that IRBMO significantly outperforms the other compared algorithms in the F-score metrics, demonstrating its efficiency and stability in classification tasks, while GOOSE, GWO and CMAES perform better, DE, EHO, GA, WOA and WSO perform moderately, and RBMO and PO perform poorly.

**Table 12:** F-score values of IRBMO vs. other binary algorithms.

Dataset		IRBMO	RBMO	DE	GA	GOOSE	GWO	WOA	PO	WSO	CMAES	EHO
BCC	Mean	0.843667	0.644	**0.903333**	0.474667	0.551	0.662667	0.607	0.265333	0.408	0.695333	0.452
	Std	0.050068	0.047677	0.071406	0.056062	0.049852	0.047411	0.126686	0.114281	0.034079	0.025425	0.032099
BCWD	Mean	0.990667	0.97	0.989667	0.981	0.986	0.988	0.978	0.98	0.99	0.998667	**1**
	Std	0.004498	4.52E-16	0.003198	0.005477	0.005632	0.005509	0.005509	3.39E-16	5.65E-16	0.003457	0
BCWP	Mean	0.878667	0.747	0.694333	0.827333	**0.92**	0.765	0.836667	0.756333	0.804667	0.840333	0.87
	Std	0.017953	0.009523	0.008172	0.007849	6.78E-16	0.039806	0.018631	0.053529	0.056369	0.025929	2.26E-16
Cleveland	Mean	0.712333	0.690333	0.709	0.695667	0.708667	0.710333	0.712667	0.663333	0.674333	0.72	**0.754333**
	Std	0.015013	0.014967	0.017489	0.01278	0.028495	0.012726	0.009803	0.015388	0.01675	0.010828	0.009714
Dermatology	Mean	**1**	0.978	**1**	**1**	**1**	**1**	0.996333	0.994667	**1**	0.996	**1**
	Std	0	0.006644	0	0	0	0	0.006149	0.006288	0	0.006215	0
DRD	Mean	0.835	0.83	0.84	0.823333	0.848	**0.868**	0.843667	0.823667	0.822333	0.82	0.83
	Std	0.014563	5.65E-16	0	0.006609	0.008052	0.007611	0.006687	0.012726	0.007279	2.26E-16	5.65E-16
Hepatitis	Mean	**0.997333**	0.890333	0.712	0.790333	0.876667	0.924	0.842333	0.766333	0.909667	0.955667	0.741
	Std	0.004498	0.009643	0.006103	0.008503	0.004795	0.005632	0.006261	0.009643	0.010981	0.00504	0.003051
ILPD	Mean	0.980333	0.964	0.996667	0.969333	**1**	0.959333	0.982333	0.972667	0.977	0.984333	0.99
	Std	0.010334	0.004983	0.005467	0.006915	0	0.005833	0.01278	0.013113	0.014179	0.009714	5.65E-16
Lymphography	Mean	0.946	0.903	0.939	0.915667	0.933333	0.949	0.909667	0.882667	0.906	**0.954**	0.904333
	Std	0.007701	0.007497	0.006618	0.006789	0.008841	0.006618	0.008087	0.004498	0.006215	0.005632	0.005683
Parkinsons	Mean	0.941333	0.885667	**0.958333**	0.909	0.947	0.947	0.920333	0.870333	0.894333	0.955667	0.941667
	Std	0.007761	0.018696	0.009499	0.012415	0.008769	0.009879	0.009994	0.015643	0.017943	0.007279	0.005307
PDC	Mean	**0.482333**	0.302333	0.301667	0.338667	0.455	0.392667	0.338333	0.324667	0.364333	0.262	0.437667
	Std	0.029674	0.046139	0.030295	0.073425	0.096874	0.059186	0.068686	0.139327	0.078945	0.106233	0.092762
Spectfheart	Mean	**1**	0.803667	0.853667	1	0.840667	0.823	0.593667	0.592333	0.868667	0.749333	0.67
	Std	0	0.061279	0.069752	0	0.056625	0.239887	0.132989	0.12381	0.127353	0.092212	3.39E-16
(W|T|L)		(4|8|0)	(0|10|2)	(3|7|2)	(2|10|0)	(3|9|0)	(2|9|1)	(0|12|0)	(0|7|5)	(1|11|0)	(1|9|2)	(3|9|0)
Mean		2.92	8.58	5.00	6.50	3.92	4.50	6.50	9.67	6.50	4.92	5.00
Rank		**1**	10	5	7	2	3	7	11	7	4	5

In summary, IRBMO, when dealing with the feature selection problem, shows excellent performance in several key evaluation indexes, such as fitness value, classification accuracy, sensitivity, specificity, the number of selected features, and F-score, etc. It not only can effectively identify the key features to reduce the complexity of the model and improve the operation efficiency, but also can strike a good balance between the precision rate and the recall rate to ensure the overall performance of the model, which shows a wide applicability and strong competitiveness.

### 5.4. Experiments comparing IRBMO with existing feature selection algorithms

Although several metaheuristic algorithms have been widely used in the feature selection field, there is still room for further optimization of their performance. In order to improve the effectiveness of feature selection, we systematically investigate the newly proposed IRBMO algorithm and make an in-depth comparison with nine algorithms that are outstanding performers in current feature selection tasks. These comparison algorithms include Binary Chimp Optimization (BCHIMP) [66], Hyper Learning Binary Dragonfly Algorithm (BDA) [67], and Binary Grey Wolf Optimizer (BGWO) [68], Binary Harris Hawk Optimization (BHHO) [69], Bare-bones Particle Swarm Optimization with Mutual Information (BPSO) [70], Binary Improved African Vulture Optimization Algorithm (AVOA) [71], Artificial Rabbits Optimization (ARO) [36], Capuchin Search Algorithm(CAPSA) [35], Electric Eel Foraging Optimization Algorithm (EEFO) [29]. These algorithms have been rigorously selected and all of them show strong competitiveness in the feature selection domain. Although IRBMO has demonstrated excellent results in feature selection tasks with meta-heuristic algorithms, we recognize that a more in-depth and detailed comparison with existing feature selection algorithms can further reveal IRBMO’s unique strengths and potential value in feature selection performance. Such a comparative analysis will not only help us understand the working principle and performance characteristics of IRBMO more comprehensively, but also provide researchers and practitioners in related fields with richer information and references to support them in making more informed decisions in feature selection tasks. Therefore, in this section, the comparative study with existing feature selection algorithms will be further deepened, with a view to revealing more comprehensively the excellent performance of IRBMO in the field of feature selection.

To ensure the fairness of the comparison and the reliability of the results, the parameters of all the algorithms were carefully tuned, and the specific configurations are detailed in Table 13. The mean and standard deviation of the fitness values were calculated for the experimental statistics, please refer to Table 14 for detailed results. The analysis of the mean reveals the average performance of the algorithms, while the standard deviation reflects the range of the fluctuation of the performance, and thus the stability of the algorithms is assessed. In addition, we visualize the experimental results through box-and-line plots (6Fig 5), which intuitively show the performance fluctuation, outlier distribution, and data distribution characteristics of the algorithms on different datasets, and provide a strong support for the analysis of stability and robustness.

**Table 13:** Parameterization of the feature selection algorithm.

Algorithm	Parameter settings
BCHIMP	*a*: Decreases linearly from 2 to 0
BDA	*Dmax* = 6; *w*: Linearly decreasing from 0.9 to 0.4; rate: Linear reduction from 0.1 to 0.05
BGWO	a: Linearly decreasing from 2 to 0
BHHO	ho =0.2
BPSO	*a1*, *a2*: Generate a random floating-point number in the range [0, 1] and multiply by the population; *c*1: Generate a random floating-point number in the range [0, 1]; *c*2:1-c1
AVOA	*P*1 = 0.6; *P*2 = 0.4; *P*3 = 0.6; *Al* = 0.8; *Be* = 0.2; *Ga* = 2.5
EEFO	*P*1, *P*2: Generate a random number between 0 and 1
CAPSA	a1, a2 =1; ρ =0.707; $P_{bf}$ =0.7; $P_{ef}$ =9; $\beta_{0}$ =2; $\beta_{1}$ =22; $\beta_{2}$ =2
ARO	*n*1, *n*2: Generate a random number between 0 and 1

**Table 14:** Fitness values of IRBMO vs. other feature selection algorithm.

Dataset		IRBMO	RBMO	IRBMO1	IRBMO2	BCHIMP	BDA	BHHO	BPSO	AVOA	EEFO	CAPSA	ARO
BCC	Mean	**0.158678**	0.18031	0.170046	0.170084	0.186635	0.175134	0.175134	0.175134	0.175134	0.162851	0.168217	0.162851
	Std	0.005427	0.018035	0.024706	0.018893	0.015926	8.47E-17	8.47E-17	8.47E-17	8.47E-17	2.82E-17	0.009525	2.82E-17
BCWD	Mean	**0.009162**	0.012804	0.009441	0.011957	0.01836	0.010525	0.013253	0.011026	0.011467	0.012083	0.013786	0.012483
	Std	0.003342	0.002566	0.000482	0.002631	0.003046	0.003192	0.002888	0.001796	0.002949	0.00077	0.00283	0.000485
BCWP	Mean	**0.021917**	0.030512	0.024168	0.02947	0.029441	0.027507	0.028731	0.026726	0.027366	0.022559	0.023303	0.022559
	Std	0.001473	0.002526	0.003409	0.001995	0.001313	0.001879	0.001	0.002182	0.001907	0	0.001319	0
Cleveland	Mean	**0.294999**	0.324574	0.312518	0.320485	0.323328	0.310959	0.312097	0.31078	0.310908	0.349164	0.362023	0.351091
	Std	0.015028	0.010988	0.015516	0.01003	0.016129	0.00069	0.002785	1.69E-16	0.000498	0.011405	0.01042	0.009214
Dermatology	Mean	0.006225	0.009187	0.00917	0.013582	0.013144	0.00946	0.007321	0.007468	0.006828	**0.005879**	0.0086	0.00623
	Std	0.002506	0.006134	0.004384	0.004344	0.005276	0.006591	0.002445	0.004343	0.000469	0.000574	0.004043	0.000465
DRD	Mean	**0.249178**	0.278723	0.276777	0.30525	0.271468	0.253214	0.258932	0.250271	0.256318	0.300152	0.306903	0.304114
	Std	0.009163	0.009288	0.010754	0.014332	0.008244	0.006004	0.004579	0.003817	0.006506	0.006387	0.006891	0.007066
Hepatitis	Mean	0.000614	0.004194	0.00372	0.022958	0.005685	0.001193	0.002088	0.000842	**0.000526**	0.003514	0.005078	0.004695
	Std	0.00048	0.009076	0.009049	0.024489	0.009096	0.000829	0.000725	0.000528	3.31E-19	0.002325	0.000922	0.001643
ILPD	Mean	**0.207832**	0.267272	0.25235	0.252057	0.259757	0.248793	0.248926	0.24886	0.249382	0.24639	0.251406	0.245751
	Std	0.010163	0.01087	0.008021	0.009724	0.011286	8.47E-17	0.000507	0.000365	0.002001	0.004252	0.007735	0.004679
Lymphography	Mean	0.095993	0.128693	0.135602	0.149816	0.12676	0.091408	0.10824	**0.086727**	0.097448	0.108777	0.119848	0.109781
	Std	0.017242	0.011476	0.014879	0.011871	0.019721	0.012312	0.010613	0.00888	0.012724	0.007485	0.007065	0.008491
Parkinsons	Mean	0.019592	0.026617	0.025859	0.025681	0.048818	0.023019	0.02735	0.023201	0.02407	**0.01903**	0.025402	0.020545
	Std	0.009075	0.008878	0.009295	0.006453	0.01626	0.000204	0.007644	0.0003	0.003633	0.002814	0.017775	0.003208
PDC	Mean	0.071582	0.099373	**0.068875**	0.092573	0.088861	0.074485	0.09797	0.088973	0.091882	0.084077	0.085266	0.085667
	Std	0.006723	0.015958	0.008162	0.007878	0.008225	0.008717	0.003159	0.005923	0.003611	0.00217	0.00285	0.001431
Spectfheart	Mean	**0.073164**	0.094463	0.089059	0.100423	0.107227	0.074174	0.116076	0.092659	0.093015	0.088065	0.091095	0.088071
	Std	0.014714	0.014962	0.015837	0.011707	0.019182	0.023089	0.013594	0.010941	0.017144	0.011385	0.009926	0.011037
(W|T|L)		(7|5|0)	(0|9|3)	(1|11|0)	(0|10|2)	(0|10|2)	(0|12|0)	(0|11|1)	(1|11|0)	(1|11|0)	(2|10|0)	(0|10|2)	(0|12|0)
Mean		1.50	9.92	6.33	9.50	10.00	4.50	7.58	4.67	5.42	4.50	7.83	5.58
Rank		1	11	7	10	12	2	8	4	5	2	9	6

And we also plotted their iterative curves for visualizing their convergence performance. And we conducted ablation experiments of IRBMO to deeply explore its internal mechanism. First, we plotted iterative graphs to visualize the convergence performance of IRBMO and its variants. In order to study the roles of elite search behavior and collaborative hunting behavior in the algorithm more clearly, we compared IRBMO1, which contains only elite search behavior, and IRBMO2, which contains only collaborative hunting behavior, respectively. Since memory storage behavior is regarded as a compensatory behavior, which is usually used in combination with elite search behavior and collaborative predation behavior, in this ablation experiment, we did not examine the effect of memory storage behavior alone, but instead focused on analyzing the independent contributions of elite search and collaborative predation behaviors to the performance of the algorithm. With this design, we are able to more accurately understand the role of each component in IRBMO and how they work together to contribute to the overall performance of the algorithm.

To comprehensively evaluate the performance of the IRBMO algorithm, we conducted an in-depth analysis in terms of both ablation experiments and comparison with existing algorithms. First, the effectiveness of the different improvement strategies in IRBMO is verified by ablation experiments. IRBMO1 introduces elite search behavior (exploration strategy), while IRBMO2 focuses on collaborative hunting behavior (exploitation strategy). Careful analysis of Table 14 shows that IRBMO achieved optimal fitness values on 7 of the 12 datasets, significantly better than IRBMO1 and IRBMO2. Specifically, IRBMO1 performs optimally on 1 dataset, but its overall performance is not inferior to that of RBMO, suggesting that while an exploration strategy alone can enhance the global search capability, the lack of support from an exploitation strategy may lead to insufficient local search capability. IRBMO2 performs optimally on 0 datasets, but its performance is slightly inferior to that of RBMO, especially on complex datasets (e.g., DRD, Lymphography), but its performance is significantly enhanced when it is combined with IRBMO1, proving the significant advantages of the exploitation strategy in terms of local search and convergence. IRBMO achieves optimal performance on most datasets by combining the exploration and exploitation strategies, verifying an effective balance between its global and local search capabilities.

Second, we compare the performance of IRBMO with a variety of existing feature selection algorithms (e.g., RBMO, BCHIMP, BDA, BHHO, etc.). The experimental results show that IRBMO performs optimally on 7 out of 12 datasets and has an average ranking of 1.5 on all datasets, which is significantly better than the other compared algorithms. In contrast, BDA and EEFO perform better with an average ranking of 4.50, but the number of times they perform optimally on the datasets is too low; BPSO, AVOA and ARO perform moderately well with average rankings of 4.67, 5.42 and 5.58, respectively; while BHHO, CAPSA, BCHIMP and RBMO perform poorly, especially on multiple datasets with high fitness values or higher fluctuations. Other existing algorithms (e.g., BCHIMP, BDA, BHHO, etc.) perform better on some datasets, but none of them is as good as IRBMO in terms of overall performance. These results fully demonstrate the strong competitiveness of IRBMO in the feature selection task.

To present the performance of the IRBMO algorithm intuitively, we adopt a box-and-line diagram for visual analysis (see Fig 7). From the figure, it can be clearly seen that the median of IRBMO algorithm is significantly less than that of other algorithms, which fully indicates that it has obvious performance advantages in most cases. Meanwhile, the IRBMO algorithm has a compact box shape and a small range of performance fluctuations, which further highlights its excellent stability. In addition, compared with other algorithms, the IRBMO algorithm has a very small number of outliers, a characteristic that reflects its strong anti-interference ability in dealing with different data sets and complex experimental conditions. IRBMO always maintains a consistent and superior performance in the face of data distribution differences and external factors.

**Fig 6:**
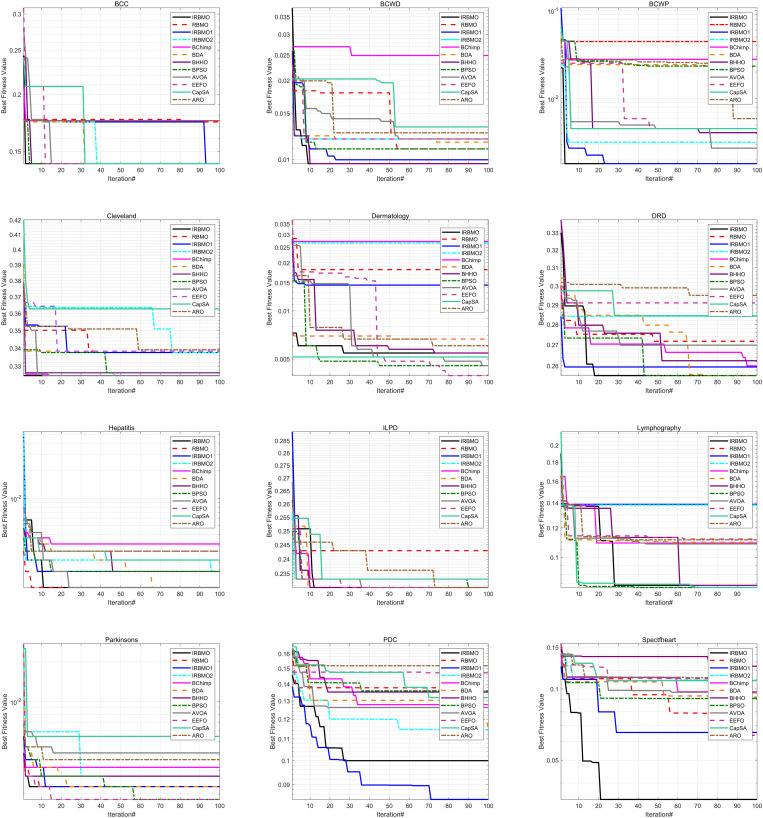
Iterative graph of IRBMO vs. other feature selection algorithms.

By analyzing the convergence curves of IRBMO with other existing feature selection algorithms in iterative Fig 6, we intuitively observe the significant advantages of IRBMO in terms of convergence speed and accuracy. On most datasets, the convergence curve of IRBMO drops rapidly at the beginning of the iteration, indicating that it is able to quickly find the search region close to the optimal solution, demonstrating an efficient global search capability. In contrast, the convergence curves of other algorithms (e.g., RBMO, BCHIMP, BDA, etc.) decline slower, especially on complex datasets (e.g., DRD, Spectfheart), and IRBMO’s convergence speed is significantly better than other algorithms. At the later stage of iteration, the convergence curve of IRBMO stabilizes at lower fitness values with less fluctuation, indicating that it is not only able to find better solutions, but also has strong stability. On the other hand, the convergence curves of other algorithms tend to stay at higher fitness values and still have large fluctuations in the late iteration, indicating that they are prone to fall into local optimization or unstable convergence. Specifically, the convergence curve of RBMO decreases slower in most datasets and the final convergence value is higher, indicating that its global and local search ability is not as good as that of IRBMO; the algorithms such as BCHIMP and BDA perform better in some datasets, but are still not as good as that of IRBMO in the whole, e.g., the convergence curve of BCHIMP fluctuates more in the late iteration period, and the convergence curve of BDA decreases slower. For example, the convergence curve of BCHIMP fluctuates more in the late iteration and the convergence curve of BDA decreases more slowly. In summary, IRBMO achieves faster and more stable convergence by combining the elite search (exploration) and collaborative hunting (exploitation) strategies to quickly explore the global search space at the beginning of the iteration, and then finely adjusts the quality of the solution through the exploitation strategy, which further proves its high efficiency and stability in the feature selection task.

### 5.5. IRBMO combined with S- and V-shaped transfer functions

While dealing with the feature selection problem in the IRBMO algorithm, since the algorithm deals mainly with sequences of continuous values, we need to convert these continuous values into binary form, i.e., 0 or 1. This requires us to employ a conversion function to achieve this conversion. Various conversion methods have been covered in previous discussions. In this section, we will further analyze a total of eight variants of transfer functions from two major families (S-form and V-form) with a view to finding algorithms that have stronger generalization capabilities and are more suitable for dealing with feature selection problems. Table 15 lists the transfer functions of these two families in detail, while Fig 8 visualizes their respective distribution curves. This in-depth analysis is intended to help us better understand, and select, the best conversion strategy for the binary representation of continuous features in IRBMO-based feature selection tasks.

**Table 15:** S and V shaped transfer functions.

S-shaped family		V-shaped family	
Name	Transfer function	Name	Transfer function
S_v1_	$TF(x)=\frac{1}{1+e^{-x}}$	V_v1_	TF(x)=|tanh(x)|
S_v2_	$TF(x)=\frac{1}{1+e^{x}}$	V_v2_	$TF(x)=|erf(\frac{\sqrt{\pi }}{2}x)|$
S_v3_	$TF(x)=\frac{1}{1+e^{-\frac{x}{2}}}$	V_v3_	$TF(x)=|\frac{x}{\sqrt{1+x^{2}}}|$
S_v4_	$TF(x)=\frac{1}{1+e^{-\frac{x}{3}}}$	V_v4_	$TF(x)=|\frac{2}{\pi }arctan(\frac{\pi }{2}x)|$

**Fig 7:**
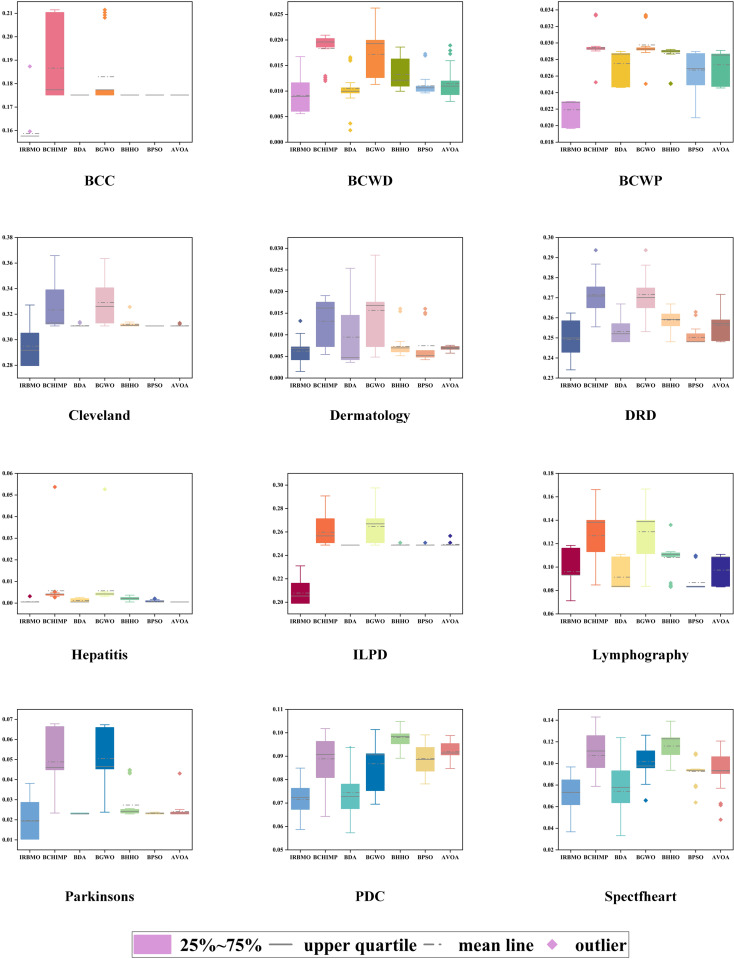
IRBMO vs. feature selection algorithm boxplot.

**Fig 8:**
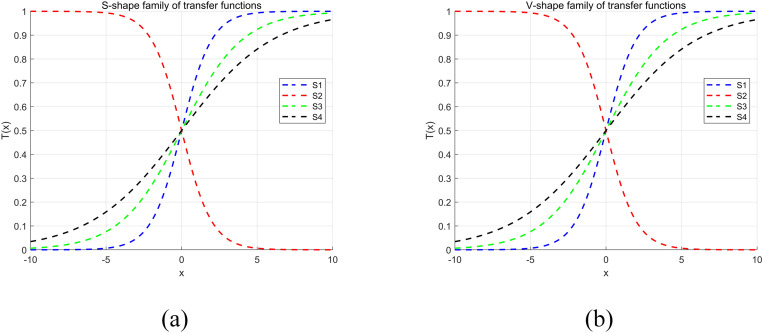
S- and V-Type transfer function diagrams.

In this section, we design eight variants of binary IRBMO (Improved Binary Multi-Objective Optimization) based on different transfer functions, specifically named S1IRBMO, S2IRBMO, S3IRBMO, S4IRBMO, V1IRBMO, V2IRBMO, V3IRBMO and V4IRBMO.

Each of these variants adopts a unique transformation mechanism designed to enhance the efficacy of feature selection. Each variant is implemented in a specific transformation format, which is designed to optimize the exploration and utilization of the feature space, thus demonstrating their respective advantages in a multi-objective optimization framework.

To ensure the accuracy and high credibility of the experimental results, we have always maintained a rigorous and meticulous scientific attitude to carry out the research work. In the experimental setup, we adopt a uniform standard, setting the number of algorithm populations strictly at 30 and the number of iterations at 100 to ensure the consistency of the experimental conditions. In order to minimize the impact of random errors on the experimental results, we adopted the strategy of repeating the experiments several times, running each algorithm independently for 30 times, and exhaustively calculating the results of the fitness runs of these 30 experiments, and taking the average as the final performance result of the algorithms under the experimental conditions. To comprehensively and accurately record and analyze the fitness values of IRBMO and its various variants on different datasets, we elaborately designed the experimental record forms to record each key data in detail. For each dataset, we not only counted the mean value of the algorithm to reflect the average performance level of the algorithm, but also calculated the standard deviation to assess the stability of the algorithm. These two metrics complement each other and together constitute a comprehensive and in-depth evaluation system of algorithm performance. In the presentation of the experimental results, we highlight the best-performing results in bold so that readers can identify at a glance the best performance of each algorithm on different datasets. Through such experimental design and data analysis methods, we strive to draw scientific and reliable research conclusions to provide strong support for the development of related fields.

As can be seen from the detailed data in Table 16, the algorithms using the simple transformation approach exhibit impressive accuracy and stability on the vast majority of datasets.

**Table 16:** IRBMO vs. variant comparison adaptation data.

Dataset	Fitness	IRBMO	S1	S2	S3	S4	V1	V2	V3	V4
BCC	Mean	**0.156954**	0.156982	**0.156954**	**0.156954**	**0.156954**	0.15778	0.15778	0.158605	0.164302
	Std	0	0.000152	0	0	0	0.004368	0.004368	0.006065	0.011055
BCWD	Mean	0.003132	0.003098	0.002922	0.003444	0.003156	0.002188	**0.001967**	0.002067	0.003794
	Std	0.001629	0.002055	0.000709	0.001129	0.000997	0.001172	0.000332	0.000458	0.002353
BCWP	Mean	0.032462	0.029946	0.03525	0.03039	0.032192	**0.024773**	0.026741	0.02553	0.032782
	Std	0.009581	0.009164	0.009112	0.010755	0.009786	0.009892	0.011103	0.00731	0.009525
Cleveland	Mean	0.314819	**0.314764**	0.321433	0.316124	0.316526	0.322005	0.32457	0.322383	0.325335
	Std	0.008102	0.007553	0.006628	0.008833	0.008227	0.008541	0.006557	0.007116	0.00385
Dermatology	Mean	0.005946	0.005685	0.010307	0.007447	0.007406	**0.004392**	0.005048	0.004614	0.009673
	Std	0.003484	0.00272	0.005265	0.004889	0.004825	0.002915	0.003504	0.002647	0.00605
DRD	Mean	0.226479	0.227428	0.227851	0.228001	0.22601	0.219629	0.221496	**0.218442**	0.228633
	Std	0.010309	0.008661	0.007851	0.009455	0.008098	0.006961	0.008154	0.003847	0.009545
Hepatitis	Mean	0.042169	0.04749	0.042194	0.045684	0.043484	0.036941	0.036345	**0.035215**	0.043269
	Std	0.009768	0.014648	0.010779	0.014159	0.012823	0.005264	0.004349	6.73E-05	0.010869
ILPD	Mean	0.20291	0.204731	**0.202**	0.202455	**0.202**	0.204015	**0.202**	0.202455	0.211853
	Std	0.003464	0.005555	0	0.002493	0	0.008817	0	0.002493	0.015697
Lymphography	Mean	0.143848	0.143996	0.142231	0.144629	0.14236	**0.13662**	0.13729	0.139092	0.143477
	Std	0.011971	0.011823	0.010969	0.01227	0.010959	0.004783	0.006626	0.009003	0.012106
Parkinsons	Mean	0.017941	0.018617	0.018767	0.01833	0.019687	0.017954	0.017289	**0.016785**	0.019057
	Std	0.002483	0.002561	0.002567	0.002528	0.002428	0.002516	0.002232	0.001786	0.003379
PDC	Mean	0.075792	0.076938	0.078553	0.076708	0.076312	**0.071672**	0.071981	0.072949	0.077026
	Std	0.004335	0.004217	0.002978	0.00364	0.005154	0.005806	0.004746	0.005228	0.002926
Spectfheart	Mean	0.066649	0.067393	0.066554	0.068705	0.066081	**0.054375**	0.054529	0.055658	0.071128
	Std	0.009073	0.008775	0.0101	0.008871	0.010814	0.009506	0.010808	0.01059	0.008366
(W|T|L)		(1|11|0)	(1|10|1)	(2|7|3)	(1|10|1)	(2|9|1)	(5|7|0)	(2|10|0)	(3|9|0)	(0|6|6)
Mean		4.67	5.75	5.50	6.00	4.92	3.08	2.92	3.08	8.17
Rank		4	7	6	8	5	2	**1**	2	9

Among these variants, the V2 variant stands out as one of the best performers with its outstanding performance. The V2 variant not only dominates in terms of average performance, but also wins wide recognition for its stable performance. This is a testament to the unique advantages of the V-shaped transfer function and its parameter configurations employed by the V2 variant in the feature selection task, allowing it to more accurately identify and retain the most valuable features for the target task. It is closely followed by the V1, V3 variant, which fails to outperform the V2 variant but still occupies the second position in the ranking. This suggests that the combination of the V1 and V3 transfer functions is somewhat complementary and can create synergy in the feature selection task, thus improving the overall performance of the algorithm. Surprisingly, however, the V4 variant is relatively low in the ranking, coming in at the ninth position. This may be related to the fact that the transfer function employed by the V4 variant and its parameter configurations are not sufficiently adapted for the current feature selection task. This also re-emphasizes the important impact of transfer function selection and parameter configuration on algorithm performance. As for the original IRBMO algorithm, it was ranked fourth in the ranking, which indicates that the algorithm itself is somewhat competitive. However, after the variants were designed by introducing S-shaped and V-shaped transfer functions, some of the variants (e.g., V2 and V1V3) showed significant improvement in performance. In addition, the four variants S4, S2, S1, and S3 constructed from the S-shaped transfer function occupied positions 5, 6, 7, and 8, respectively, in the ranking. Although these variants fail to outperform the variants constructed by V-shaped transfer functions, they still show some stability and competitiveness. This further demonstrates the potential of transfer function diversity for algorithmic performance improvement. It is worth noting that while these algorithmic variants showed impressive accuracy and stability on most datasets, performance fluctuations were observed on three specific datasets. Such fluctuations may be related to factors such as the completeness of the dataset, noise level, and distributional characteristics. This reminds us that we need to be more careful in selecting and handling datasets in subsequent studies to ensure stable and reliable algorithm performance.

In summary, the IRBMO algorithm and its variants constructed via S-shaped and V-shaped transfer functions demonstrate rich performance and in-depth analytical value on the task of medical data feature selection. These results provide useful references and insights for the optimization and improvement of subsequent algorithms.

## 6. Conclusion

To deeply explore the performance of the IRBMO algorithm in the field of feature selection for medical data, we have carefully designed and implemented an experiment covering 12 medical datasets with different complexities. The experiment aims to reveal the unique strengths and potential weaknesses of each algorithm in multiple dimensions, with a special focus on the specific contribution of the IRBMO algorithm in improving classification performance. To this end, a comprehensive comparison between IRBMO and the base RBMO algorithm was conducted to accurately assess its gains in feature selection.

During the experiments, we chose the classical KNN (K Nearest Neighbors) classifier as the baseline model. The wide acceptance and performance stability of KNN make it an ideal experimental tool, while it can clearly reflect the direct impact of feature selection on classification results. The experimental results in Table 17 show that the IRBMO algorithm improves the classification performance by 43.89% on average compared to the baseline model. This significant increase not only verifies the excellent performance of IRBMO in feature selection, but also demonstrates its strong potential in optimizing the overall classification performance.

**Table 17:** wIRBMO feature selection algorithm improving rate.

Dataset	RBMO		IRBMO		Improvement(%)	
	Mean	Std	Mean	Std	Mean	43.89
BCC	0.209143	0.014925	0.158678	0.005427	24.13%	
BCWD	0.013285	0.003015	0.009162	0.003342	31.03%	
BCWP	0.12178	0.001652	0.021917	0.001473	82.00%	
Cleveland	0.327749	0.002705	0.294999	0.015028	9.99%	
Dermatology	0.090265	0.004511	0.006225	0.002506	93.10%	
DRD	0.281317	0.005649	0.249178	0.009163	11.42%	
Hepatitis	0.08957	0.000865	0.000614	0.00048	99.31%	
ILPD	0.301455	0.001829	0.207832	0.010163	31.06%	
Lymphography	0.16949	0.01531	0.095993	0.017242	43.36%	
Parkinsons	0.028418	0.001634	0.019592	0.009075	31.06%	
PDC	0.113844	0.009212	0.071582	0.006723	37.12%	
Spectfheart	0.109387	0.017265	0.073164	0.014714	33.12%	

Overall, the IRBMO algorithm performs well in the medical data feature selection task and significantly improves classification performance. This finding lays an important foundation for subsequent algorithm optimization and practical application, and also points out the direction for further exploration and improvement of feature selection strategies, providing strong support for advancing the development of medical data analytics.

This study has some limitations in the following aspects: first, the relatively small number of features and limited complexity of the dataset used may not fully cover the challenges of feature selection for real-world high-dimensional and nonlinear data; second, only the basic KNN classifier was used in the experiments, which is simple and easy to use, but may not be able to show the full potential of the feature selection algorithms; in addition, the design of the algorithm mainly focuses on the balance between development and exploration, the range of parameter tuning may be somewhat limited, and the tuning process of hyperparameters is not described in detail, which may have some impact on the robustness and reproducibility of the results. In order to further enhance the depth and breadth of the study, future work may consider introducing more high-dimensional datasets, combining multiple advanced classifiers, and further refining the parameter tuning strategy, so as to more comprehensively validate the performance of the algorithm and its scalability in practical applications.

In the wave of digital transformation, data has become the core driving force for social change and life innovation, and its potential information value is immeasurable. Especially in the healthcare field, data is not only an important resource for life safety, but also a source of wisdom for disease research, diagnosis and treatment decisions, and prevention strategies. However, with the exponential growth of data size, complexity and diversity, data processing and analysis are facing unprecedented challenges. The “dimensionality catastrophe” brought by high-dimensional data further aggravates the difficulty of analysis and adds obstacles to data insight. In this context, feature selection technology, as an important part of data preprocessing, is becoming more and more critical. By accurately identifying core features and reducing data dimensionality, feature selection technology significantly improves analysis efficiency and accuracy.

In this study, an innovative “Multi-behavioral Enhanced IRBMO Feature Selection Algorithm” (IRBMO) is proposed to address the key challenges in feature selection of medical data. The algorithm is based on the red-billed blue magpie optimization framework, and incorporates several innovations such as elite search strategy, collaborative hunting mechanism and memory storage strategy, which successfully realize the efficient optimization of the feature selection problem. These designs not only significantly improve the computational efficiency and selection accuracy of the algorithm, but also enhance its robustness and adaptability. In a series of tests on medical datasets of different sizes and dimensions, IRBMO demonstrates excellent performance. To comprehensively verify the performance of IRBMO, we conducted comparative experiments with the binary versions of other meta-heuristic algorithms as well as existing feature selection algorithms, and used a variety of evaluation metrics, such as fitness value, classification accuracy, sensitivity, specificity, number of selected features, F-scores, rank test, and Fiederman’s test, to perform a comprehensive evaluation. In addition, we constructed eight IRBMO variants with eight transfer functions to deeply explore their potentials in feature selection of medical data, and successfully constructed the V2IRBMO variant with better performance. Experimental results show that IRBMO achieves an overall performance improvement of 43.89% compared to the original RBMO algorithm. In addition, compared with mainstream optimization algorithms and existing feature selection methods, IRBMO has significant advantages in terms of accuracy, stability and efficiency.

Although this study has achieved important results in the field of feature selection, there are still several directions worth exploring. For example, although the packing method, as one of the high-performance feature selection methods, performs well on high-dimensional datasets, its computational complexity increases significantly with the growth of data size, which puts high demands on computational resources and time. Therefore, future research will focus on exploring the deep integration of the packing method with the filtering method and the embedded method, in order to fully utilize their respective advantages and balance the relationship between high accuracy and low complexity. In addition, we plan to introduce an adaptive parameter optimization strategy so that the algorithm can dynamically adjust the parameters according to the characteristics of different datasets, further enhancing its versatility and practicality. In the future, we will also expand the application areas of IRBMO algorithm, such as practicing it in complex data scenarios such as image processing and natural language processing. The complex data structure and strong feature correlation in these fields provide a broad stage for IRBMO to show its potential. Meanwhile, we will also explore the performance of IRBMO in prediction tasks, with a view to providing innovative solutions for more practical application scenarios.

In summary, this study provides an efficient and innovative solution in the field of feature selection by proposing the IRBMO algorithm. The algorithm breaks through the limitations of traditional methods and achieves significant improvement in performance, stability and applicability, showing a wide range of application prospects. In the future, we will continue to deepen our research on IRBMO, optimize its performance, promote its practical application in more fields, and contribute more wisdom and strength to the innovative development of data science.
